# De Novo Design Strategies of Nanomedicines for Diabetic Wound Microenvironment Remodeling

**DOI:** 10.34133/bmef.0309

**Published:** 2026-09-16

**Authors:** Junyi Zhang, Shuo Li, Nina Liu, Zhenyu Wu, Gang Liu, Chao Liu

**Affiliations:** ^1^ State Key Laboratory of Vaccines for Infectious Diseases, Xiang An Biomedicine Laboratory, and Fujian Provincial Key Laboratory of Innovative Drug Target Research, School of Pharmaceutical Sciences, Xiamen University, Xiamen 361102, China.; ^2^ State Key Laboratory of Vaccines for Infectious Diseases, Xiang An Biomedicine Laboratory, Fujian Engineering Research Center of Molecular Theranostic Technology, School of Public Health, Xiamen University, Xiamen 361102, China.; ^3^ State Key Laboratory of Cellular Stress Biology, Innovation Center for Cell Biology, School of Life Sciences, Xiamen University, Xiamen 361102, China.; ^4^ Shenzhen Research Institute of Xiamen University, Shenzhen 518000, China.

## Abstract

Diabetic wounds are arrested by a hostile microenvironment of hyperglycemia, inflammation, infection, and hypoxia. Nanomaterials offer solutions, yet existing reviews lack a systematic framework linking their actions to the interconnected pathological network. Here, we present a pathology network-guided analysis. We delineate how engineered nanosystems from metal-based antimicrobials to glucose-responsive nanoreactors and extracellular vesicles can intercept multiple drivers, including advanced glycation end-products, oxidative stress, biofilms, and immune dysregulation. Critically, we propose a temporally phased roadmap that prioritizes neutralization of upstream drivers (hyperglycemia and biofilms) before downstream interventions (reactive oxygen species scavenging, immunomodulation, and angiogenesis), avoiding premature single-target therapy. Beyond efficacy, we critically address design trade-offs: we examine the evidence for metal nanomaterial selectivity toward bacteria versus stressed host cells, confront bacterial adaptive resistance (efflux pumps and biofilm reinforcement), and scrutinize the trade-off, manufacturing scalability, long-term biosafety, and regulatory ambiguities. By integrating mechanistic insights with clinical scalability and regulatory feasibility, we provide a conceptual framework to guide the rational design of next-generation nanomedicines that prioritize genuine synergy over redundant integration, and outline a roadmap for translation.

## Introduction

Diabetic wounds (DWs) represent a severe and escalating complication of diabetes mellitus, imposing a substantial burden on global healthcare systems and substantially compromising patient quality of life [1]. With global diabetes prevalence projected to rise to 643 million by 2030 and 783 million by 2045, the management of associated complications, particularly chronic nonhealing ulcers, has become an imminent health crisis [2]. Despite the availability of standard clinical interventions, ranging from systemic glycemic control and surgical debridement to infection management, the treatment of DWs remains fraught with challenges [3–5]. Under physiological conditions, cutaneous healing follows a well-orchestrated sequence of hemostasis, inflammation, proliferation, and remodeling [6]. However, in diabetes, these chronic ulcers frequently fail to progress through these essential orderly phases of tissue repair, stalled by a hostile microenvironment characterized mainly by chronic hyperglycemia, which results in complications including bacterial infections and oxidative stress [7].

Current therapeutic modalities, while foundational to wound care, are often constrained by limitations in bioavailability and tissue penetration within the complex diabetic niche. For instance, systemic blood glucose control is essential but insufficient to solely normalize the local wound milieu and is burdened by risks of hypoglycemia [8]. Similarly, the empiric use of topical antibiotics has catalyzed the emergence of MDR bacterial strains. Surgical debridement, a cornerstone of DW management, effectively removes necrotic tissue and reduces bacterial burden; however, when performed without appropriate indications, thorough vascular assessment, or meticulous technique, it risks disrupting viable tissue and precipitating more severe wound infections [9]. Consequently, the difficulty in simultaneously controlling infection, ischemia, and chronic inflammation with conventional agents alone frequently results in delayed recovery and, in severe cases, limb amputation [10,11].

Advances in understanding the mechanisms governing tissue regeneration and the specific hurdles of the diabetic microenvironment have driven the development of nanotechnology-based strategies. The unique chemical, mechanical, and biological properties that materials exhibit at the nanoscale provide innovative solutions to address the limitations of conventional wound care [12]. Nanomaterials, by virtue of their high surface-to-volume ratio, tunable physicochemical properties, and capacity for multifunctional integration, have emerged as a uniquely suited platform to reprogram the pathological wound milieu [13]. These applications of nanotechnology are based on the ability of nanomaterials to interact with cellular components and trigger specific regenerative signals, either through their intrinsic physicochemical cues or by serving as intelligent delivery vehicles for therapeutic agents [14,15]. Unlike traditional dressings, engineered nanoplatforms can achieve targeted bioavailability and spatiotemporally controlled release of bioactive cargos, thereby enabling precise modulation of the complex, interdependent barriers that characterize DW healing [16].

Despite the rapid expansion of the literature in this field, existing reviews have largely adopted a function-centric approach, cataloguing nanomaterial strategies according to their individual therapeutic actions, such as antibacterial, antioxidant, or pro-angiogenic effects, and thereby treating these mechanisms as parallel rather than interconnected pathways. Consequently, several critical dimensions remain insufficiently addressed. First, the complex, self-reinforcing network of pathological factors in the DW microenvironment in which hyperglycemia, oxidative stress, biofilm infection, and immune dysregulation reciprocally amplify one another is frequently described in isolation, without a systematic analysis of how nanomaterials can simultaneously intercept multiple nodes of this network to achieve synergistic therapeutic benefits. Second, although numerous nanosystems have demonstrated encouraging preclinical results, the translational barriers that impede their clinical adoption, including manufacturing scalability, long-term biosafety, and regulatory ambiguity, are often discussed superficially or as an afterthought, rather than being integrated into the conceptual framework of nanomaterial design. Third, the field lacks a critical appraisal of the prevailing design paradigm that increasingly favors multifunctional integration; the attendant complexity–cost trade-offs and the empirical question of whether more functions are necessarily better have yet to be rigorously examined.

**Fig. 1.**
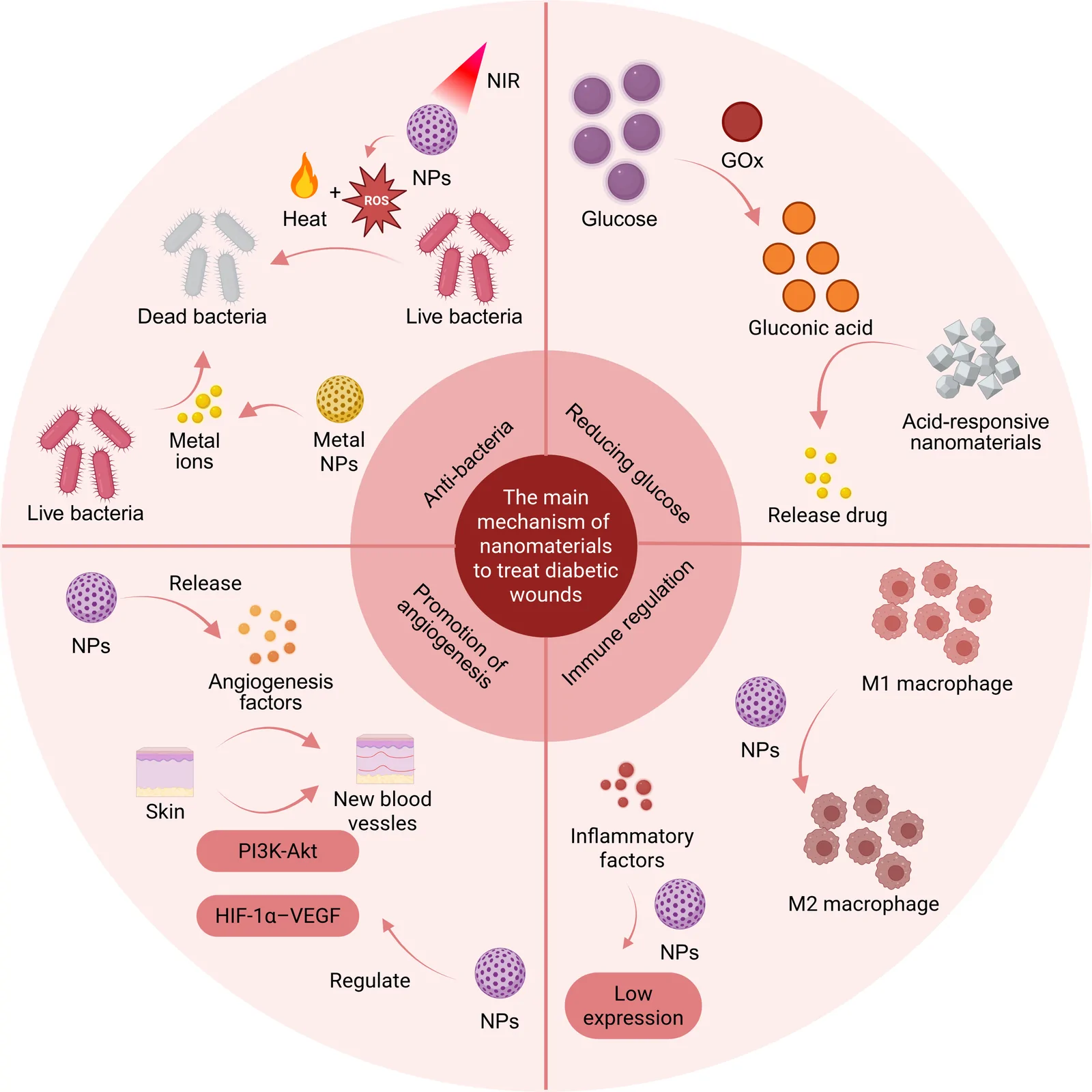
The main mechanisms of nanoparticles to treat DWs. The mechanisms include killing bacteria by PTT and PDT and releasing metal ions, lowering blood sugar levels by GOx-based systems, promoting angiogenesis by delivering angiogenesis and activating related signaling pathways, and reducing the levels of inflammatory factors through modulation of macrophage phenotype and down-regulation of inflammatory factors.

Here, we provide a systematic overview of the current landscape of DW management and discuss how integrating nanotechnology approaches can specifically remodel the wound microenvironment to restore healing competence (Fig. 1). A literature search was conducted using the PubMed database, covering English-language articles published up to August 2026. As for DW pathological mechanisms, the key search term was “mechanisms of diabetic wounds”. Articles were included if they investigated or described pathological mechanisms of DWs. As for clinical treatments of DWs, the key search term was “clinical treatment of diabetic wounds”. Articles were included if they described clinical treatment of DWs. As for nanomaterial-based strategies for exceeding DWs healing promoted by others, the key search term was “diabetic wound healing”. Articles were included if they investigated nanotechnology-based strategies for modulating the DW microenvironment; studies not directly addressing nanomaterial-mediated wound repair were excluded. Representative preclinical and clinical studies were selected based on their methodological quality, translational relevance, and ability to illustrate the core mechanisms discussed in this review. We highlight nanotechnology’s transformative potential to target the core pathological drivers of chronic wounds, ranging from the elimination of drug-resistant bacteria via photothermal therapy (PTT), photodynamic therapy (PDT), and metal ion release, and the reduction of local blood glucose levels using glucose oxidase (GOx)-based systems, to the promotion of neovascularization through the regulation of angiogenic signaling pathways such as the hypoxia-inducible factor-1α (HIF-1α)/vascular endothelial growth factor (VEGF) axis. Furthermore, we examine immunomodulatory approaches designed to resolve chronic inflammation by reprogramming macrophage phenotypes from a pro-inflammatory to a regenerative state (often described as the M1-to-M2 transition, though this binary framework oversimplifies the heterogeneous and dynamic phenotypes of macrophages during wound healing, and M2 polarization is not universally beneficial) and suppressing pro-inflammatory mediators. This review systematically examines how nanomaterials can modulate the wound microenvironment to promote repair and concludes by discussing key future prospects, including the design of feedback-responsive intelligent systems and the integration of artificial intelligence (AI), as well as the translational challenges in manufacturing and regulation that will shape the future of nanotechnology for DWs care.

Innovatively, we propose a “de novo design” paradigm for nanomedicines targeting DWs. In contrast to conventional formulation strategies that primarily focus on optimizing the physicochemical properties of known carriers or engineering stimuli-responsive release of existing drugs, our de novo framework begins with a systems-level deconstruction of the pathological network. It mandates that the design logic be derived a priori from the causal architecture of the disease—specifically, from the hierarchical stratification of pathological factors and their temporal evolution during wound healing. This paradigm shift entails 3 core principles: (a) network-driven targeting, which prioritizes the neutralization of upstream drivers before addressing downstream sequela; (b) temporal phasing, which sequences therapeutic actions—anti-biofilm, followed by reactive oxygen species (ROS) scavenging and immunomodulation, and subsequently pro-angiogenesis—to align with the dynamic healing cascade and avoid premature or counterproductive interventions; and (c) synergy by design, which distinguishes genuine functional synergy from redundant functional stacking, guided by the quantitative hallmarks of a successfully remodeled microenvironment. Thus, de novo design is not merely an exercise in creating new materials; it is a systematic approach to engineering a programmed, context-aware therapeutic sequence that dismantles the disease network at its roots.

Given the exponential growth of the literature, the necessity of yet another review on nanomaterials for DWs may reasonably be questioned. The justification, however, lies not in the enumeration of new studies, but in a fundamental paradigm shift that has been conspicuously absent from previous syntheses. Existing reviews, although comprehensive in cataloguing the therapeutic functions of nanomaterials, including antibacterial, antioxidant, pro-angiogenic, and immunomodulatory, have largely treated these functions as parallel, independent modules. This function-centric approach, while informative, inadvertently perpetuates a “function-stacking” mindset, in which the implicit assumption is that more functions equate to superior therapeutic outcomes. Critically, this paradigm overlooks a central biological reality: the pathological factors of DWs are not isolated but are organized into a hierarchical, self-reinforcing network, and the temporal sequence of intervention is as decisive as the choice of therapeutic agent. This review is therefore motivated by a pressing conceptual gap: the absence of a systems-level framework that specifies when, in what sequence, and why specific nanomaterial functions should be deployed.

## Pathological Mechanisms Underlying Impaired Healing in DWs

**Fig. 2.**
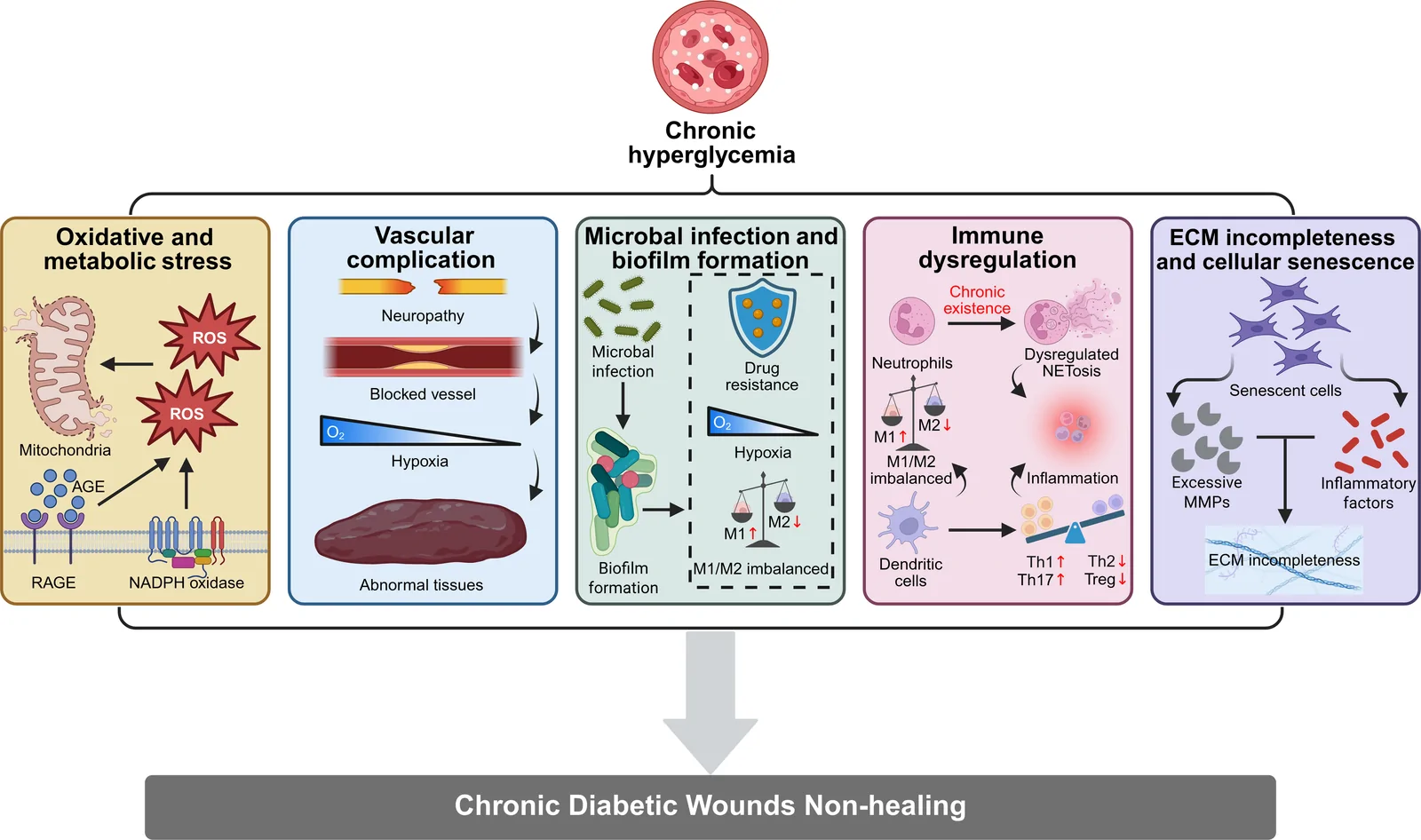
Complex pathological mechanisms underlying nonhealing chronic diabetic wounds. Chronic hyperglycemia acts as the core trigger, propagating a cascade of interconnected pathologies. The diagram delineates 5 main axes of dysfunction: (a) oxidative and metabolic stress mediated by the AGE/RAGE axis, mitochondrial dysfunction, and NADPH oxidase-induced ROS production; (b) vascular complications characterized by neuropathy, microvascular occlusion, and severe local hypoxia; (c) microbial infection and biofilm formation, which foster drug resistance, further hypoxia, and macrophage M1/M2 polarization imbalance; (d) immune dysregulation involving dysregulated NETosis, aberrant dendritic cell activation, and altered T cell polarization; and (e) ECM incompleteness and cellular senescence, where senescent cells secrete excessive MMPs and inflammatory cytokines, leading to ECM degradation. Collectively, these synergistic factors perpetuate oxidative stress, a chronic inflammatory state, and a hypoxic microenvironment, fundamentally disrupting the tissue repair sequence and culminating in nonhealing chronic diabetic wounds.

The pathogenesis of nonhealing DWs arises from a complex, self-perpetuating network of interconnected abnormalities rather than from isolated pathological pathways. At the core of this network lies chronic hyperglycemia, which serves as the primary driver initiating and sustaining a cascade of systemic and local derangements [7]. However, hyperglycemia alone does not fully account for the refractory nature of DWs; rather, its deleterious effects are profoundly amplified by reciprocal interactions with vascular insufficiency, immune dysfunction, extracellular matrix (ECM) remodeling defects, and cellular senescence. Collectively, these interdependent factors create a hostile microenvironment that perpetuates a persistent inflammatory and nonregenerative state, trapping the wound in a chronic nonhealing cycle (Fig. 2).

### Oxidative stress and metabolism dysregulation

Sustained hyperglycemia fundamentally reprograms cellular metabolism and signaling in the wound milieu. Excess glucose is diverted into alternative metabolic pathways, including the polyol, hexosamine, and protein kinase C pathways, which generate toxic intermediates and promote the accumulation of advanced glycation end-products (AGEs) [17]. The binding of AGEs to their receptor (RAGE) initiates a signaling cascade that markedly induces oxidative stress, disrupts mitochondrial function, impairs energy production, and triggers apoptosis in critical wound-resident cells such as fibroblasts, keratinocytes, and endothelial cells [18]. This metabolic and oxidative injury depletes the cellular workforce essential for granulation tissue formation and re-epithelialization.

Beyond AGE-RAGE signaling, hyperglycemia directly drives excessive production of ROS through mitochondrial dysfunction and NADPH oxidase activation [19]. The resulting oxidative stress overwhelms endogenous antioxidant defenses, damaging cellular lipids, proteins, and DNA [20]. Importantly, ROS function not merely as damaging by-products but also as signaling molecules that amplify pro-inflammatory pathways, notably via NF-κB activation, thereby perpetuating a vicious cycle of inflammation and oxidative injury [21]. This metabolic–oxidative–inflammatory axis constitutes the biochemical foundation upon which other pathological processes are built.

### Vascular complications caused by neuropathy

The DW does not exist in isolation; it is profoundly shaped by the systemic vascular and neurological complications of diabetes. Peripheral neuropathy, which affects up to 50% of patients with long-standing diabetes, leads to sensory, motor, and autonomic dysfunction [22]. Loss of protective sensation predisposes the wound to repetitive trauma and pressure injury, while autonomic neuropathy impairs sweat gland function, resulting in dry, fissured skin that serves as a portal for bacterial entry [22,23]. More critically, autonomic denervation compromises vasomotor regulation and local blood flow, directly contributing to tissue ischemia [24].

Concurrently, peripheral arterial disease (PAD), driven by accelerated atherosclerosis and medial arterial calcification, reduces macrovascular perfusion to the lower extremities [25]. The combination of PAD and microvascular dysfunction, arising from hyperglycemia-induced endothelial injury, basement membrane thickening, and reduced capillary density, creates a state of profound tissue hypoxia and nutrient deprivation within the wound bed. This hypoxic milieu not only stalls energy-dependent regenerative processes but also stabilizes HIF-1α [26]. Although HIF-1α stabilization is initially intended to trigger compensatory angiogenesis, it becomes dysregulated in the diabetic context, leading to aberrant and insufficient neovascularization [26,27]. Furthermore, impaired perfusion limits the delivery of systemic antibiotics and endogenous immune cells to the wound site, further compromising infection control [24,28].

### Microbial infection and biofilm formation

The glucose-rich, hypoxic, and ischemic environment of DWs provides a favorable niche for pathogenic bacterial proliferation [29]. Importantly, bacterial colonization is not merely a secondary complication but actively drives healing failure. Pathogens secrete virulence factors such as lipopolysaccharide (LPS) from gram-negative bacteria and lipoteichoic acid (LTA) from gram-positive bacteria. These pathogen-associated molecular patterns engage pattern recognition receptors on immune cells, activating NF-κB signaling and driving the excessive transcription of pro-inflammatory cytokines, including interleukin-1α (IL-1α), IL-6, IL-8, and tumor necrosis factor-α (TNF-α) [30]. This sustained, hyperactive inflammatory response prevents the wound from transitioning into the proliferative and remodeling phases.

Critically, pathogenic bacteria in DWs frequently organize into biofilms which structured communities embedded within a self-produced extracellular polymeric matrix [29]. Biofilms not only shield bacteria from host immune defenses and antimicrobial agents but also act as a diffusion barrier that restricts drug penetration. Moreover, biofilms perpetuate local hypoxia by consuming oxygen and nutrients, and they release persistent pro-inflammatory stimuli that sustain M1-dominant macrophage polarization and chronic inflammation [31]. The presence of biofilms therefore constitutes a physical, biochemical, and immunological barrier that must be overcome for healing to proceed.

### Immune dysregulation

The immune response in DWs is profoundly and persistently dysregulated, extending well beyond the widely recognized M1/M2 macrophage imbalance. While macrophages serve as central orchestrators, other immune cell populations also contribute critically to the nonhealing state [32].

Neutrophils are the first responders to skin injury, rapidly infiltrating the wound to phagocytose pathogens and releasing neutrophil extracellular traps (NETs) that entrap bacteria [33]. In DWs, however, neutrophils exhibit prolonged survival and dysregulated NETosis. Excessive NET formation, together with elevated ROS and proteases, perpetuates tissue damage, promotes microvascular thrombosis, and fuels chronic inflammation [33]. Moreover, the failure of timely neutrophil clearance and efferocytosis, the phagocytic removal of apoptotic neutrophils by macrophages, further sustains the inflammatory milieu and impairs the transition to resolution [34].

T lymphocytes also play critical roles. In diabetes, CD4^+^ T helper subsets are skewed: Th1 and Th17 cells promote sustained inflammation, whereas regulatory T cells (Tregs) and Th2 cells, which facilitate resolution and tissue repair, are relatively diminished [35]. This imbalanced Th1/Th2 or Th17/Treg ratio skews the microenvironment toward persistent inflammation. Dendritic cells, although less extensively studied in DWs, are potent antigen-presenting cells that shape T cell responses and can influence macrophage polarization through secreted cytokines [36].

### ECM dysregulation and cellular senescence

The ECM is not merely a passive scaffold but a dynamic, bioactive compartment that instructs cell behavior through biochemical and biophysical cues [37]. In DWs, ECM homeostasis is profoundly disrupted. Hyperglycemia promotes nonenzymatic glycation of matrix proteins, which alters their structural conformation and increases their resistance to proteolytic degradation [38]. Concurrently, the balance between matrix metalloproteinases (MMPs) and their tissue inhibitors is shifted toward excessive proteolysis, driven by oxidative stress and chronic inflammation [39]. The resulting fragmented, disorganized ECM fails to provide adequate structural support for cell migration and is unable to present growth factors in their bioactive conformation. This loss of ECM integrity also impairs mechanotransduction, further compromising the function of fibroblasts and keratinocytes.

Cellular senescence has recently emerged as a key contributor to the pathogenesis of DWs [40]. Hyperglycemia, oxidative stress, and persistent inflammatory signals induce premature senescence in fibroblasts, endothelial cells, and keratinocytes. Senescent cells progressively accumulate in chronic wounds and acquire a senescence-associated secretory phenotype (SASP), which is characterized by the secretion of pro-inflammatory cytokines, MMPs, and growth factors [41]. Paradoxically, the SASP reinforces the inflammatory and proteolytic milieu while simultaneously impairing regenerative capacity. Furthermore, the SASP promotes senescence in neighboring cells through paracrine mechanisms, establishing a self-amplifying loop that perpetuates tissue dysfunction. Consequently, eliminating senescent cells (senolysis) or pharmacologically modulating their secretory profile has emerged as a promising therapeutic frontier.

### The integrated disease network

Critically, the pathological processes described above do not operate in isolation or in a simple linear sequence; rather, they constitute a highly interconnected, mutually reinforcing network. Hyperglycemia drives the formation of AGEs and the overproduction of ROS, which together impair vascular function and promote tissue hypoxia and ischemia. Hypoxia, in turn, stabilizes HIF-1α but also activates inflammatory pathways and facilitates biofilm formation. Biofilms perpetuate infection and chronic inflammation, further compromising vascular perfusion and recruiting dysfunctional immune cells. Dysregulated immune cells secrete proteases and pro-inflammatory cytokines that degrade the ECM and induce cellular senescence. Senescent cells, through their SASP, amplify inflammation and ECM degradation, while the degraded ECM fails to support proper immune cell trafficking and angiogenesis. This intricate web of positive feedback loops traps the wound in a nonhealing state, in which each factor exacerbates the others and no single intervention is sufficient to fully restore homeostasis.

## Clinical Therapy for DWs

**Fig. 3.**
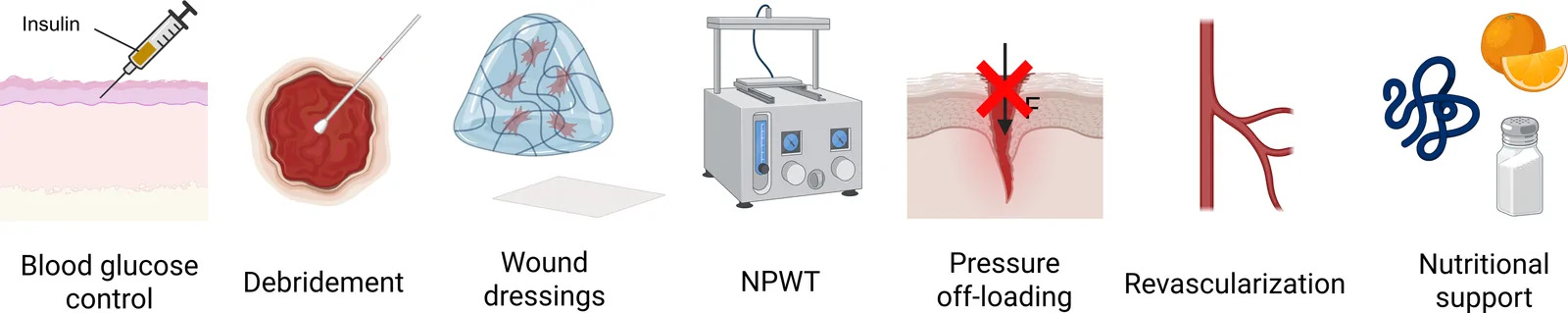
Current methods of treating DWs. The methods include controlling blood glucose, performing debridement to remove necrotic tissues, using wound dressings like hydrogel and film, NPWT that removes bacteria and other foreign substances through negative pressure, avoiding pressure on wound area, vascular assessment and revascularization, and providing enough nutrition including proteins, vitamins, and mineral salt.

Conventional management of DWs relies on a multimodal approach centered on systemic blood glucose control, wound debridement, topical antibiotic administration, etc. While these interventions target key pathological features of chronic diabetic ulcers, they are often insufficient to overcome the complex biological barriers to healing, frequently resulting in delayed recovery, high recurrence rates, and, in severe cases, limb amputation. The common clinical methods for treating DWs are shown in Fig. 3.

### Blood glucose control

Hyperglycemia lies at the core of impaired wound healing in diabetes, disrupting cellular metabolism, angiogenesis, and immune function. Consequently, stringent glycemic control remains a foundational therapeutic pillar. Subcutaneous insulin administration is the most commonly employed strategy for achieving this goal. Insulin binds to the insulin receptors on the cell surface in the body, promoting the uptake and utilization of glucose by the cells, thereby effectively lowering blood sugar levels [42]. However, this approach is burdened by several limitations. Firstly, insulin is only effective in controlling blood sugar levels for type I diabetes. For type II diabetes, the body’s sensitivity to insulin decreases, resulting in poor blood sugar control [43]. Moreover, repeated injections can lead to lipohypertrophy—localized fibrofatty nodules at injection sites—that not only cause discomfort but also alter insulin absorption kinetics, undermining therapeutic efficacy. More critically, systemic insulin therapy may carry a certain degree of safety concerns, including the risk of life-threatening hypoglycemia and hypersensitivity reactions. As for those suffering from type II diabetes, potential exacerbation of long-term cardiovascular complications caused by insulin contains a high probability [44]. These adverse effects often compromise patient adherence, thereby diminishing the overall effectiveness of metabolic management in wound healing.

In addition to insulin therapy, oral glucose-lowering agents play a key role in glycemic management [45]. For example, metformin promotes the phosphorylation of AMP-activated protein kinase (AMPK), which indirectly suppresses hepatic gluconeogenesis and thereby helps to control blood glucose levels [46]. However, the oral administration of metformin is associated with adverse effects, including vitamin B12 deficiency, gastrointestinal discomfort, and diarrhea [47,48]. Notably, the onset time and bioavailability of oral hypoglycemic drugs are comprehensively modulated by multiple key factors, including pharmaceutical formulation characteristics, gastrointestinal absorption efficiency, in vivo pharmacokinetic profiles, and the inherent pharmacological action mechanism of the drugs [49,50]. These multidimensional factors collectively determine the overall glycemic regulation efficacy of oral antidiabetic medications in clinical practice.

### Debridement and topical antibiotics

Owing to oxidative stress and other pathological factors, necrotic tissue inevitably accumulates in DWs. If not promptly removed, this necrotic burden impedes the formation of granulation tissue, provides a favorable environment for bacterial proliferation, and ultimately precipitates wound infection. Therefore, timely debridement to remove necrotic and devitalized tissue is essential for establishing a viable wound bed that supports re-epithelialization and granulation [4,51]. Debridement confers several well-established benefits: it reduces the bacterial bioburden, removes the physical barrier imposed by slough and eschar, exposes the underlying viable tissue to oxygen and topical therapeutics, and, by provoking a controlled acute inflammatory response, stimulates the local release of endogenous growth factors. Debridement techniques are broadly categorized as surgical, autolytic, enzymatic, mechanical, and biological [52]. The choice of debridement modality is guided by wound characteristics, the extent of necrosis, and patient-specific factors. Indications for debridement include the presence of nonviable tissue (eschar or slough), clinical signs of infection, peri-wound inflammation, and failure of wound progression despite adequate off-loading and moisture balance. Surgical debridement, in particular, is warranted in cases of extensive necrosis, deep abscess formation, or spreading cellulitis that require rapid source control [53]. It is also a necessary prerequisite before the application of advanced therapies such as negative-pressure wound therapy or skin grafts.

However, debridement itself is not without drawbacks. Although therapeutically necessary, it can inadvertently enlarge the wound defect and disrupt nascent tissue architecture, thereby increasing susceptibility to secondary infection [53]. This concern is particularly pronounced with surgical debridement, whose invasive nature also contributes to patient discomfort and poor compliance. Notably, biological debridement using maggots effectively removes necrotic tissue, as the larvae selectively feed on devitalized tissue, which seems painless [54,55]. Nevertheless, its clinical application is constrained by inconsistent availability, variable patient acceptance, occasional discomfort, specific contraindications, and heterogeneous clinical evidence [56–60]. Therefore, major contraindications include severe ischemia without prior or concurrent revascularization, as debriding an under-perfused wound may extend necrosis; unstable cardiovascular or metabolic status that precludes anesthesia; and, in the case of biological debridement, patient allergy to maggot secretions or wounds with exposed major vessels or nerves. In patients with critical limb ischemia, debridement should be deferred until revascularization is performed to ensure adequate oxygen and nutrient supply for subsequent healing.

In infected wounds, debridement is typically combined with topical antibiotic application to suppress bacterial colonization [61]. The most common bacterial cause of DWs infection is *Staphylococcus aureus*, which is often treated by oral amoxiclav In addition, fungal infections represent an under-recognized yet clinically marked contributor to DW complications [61]. Superficial fungal infections, including tinea pedis and onychomycosis, directly increase the risk of developing diabetic foot ulcers (DFUs) and subsequent secondary infections in patients with diabetes [62]. For fungal infections associated with DFUs, azole antifungal agents, such as fluconazole, are commonly employed [63]. Nevertheless, the widespread and often empiric use of topical antibiotics has accelerated the emergence of MDR bacterial strains, severely limiting future therapeutic options and complicating infection control in chronic wounds [64].

### Wound dressings

Wound dressings are widely applied to the surface of DWs to provide a physical barrier against airborne microorganisms and to create an environment conducive to healing. Historically, simple materials such as gauze and bandages were employed primarily for their capacity to absorb wound exudate [65]. However, these conventional dressings lack intrinsic pharmacological activity. Moreover, though modern gauze can be impregnated with antimicrobial agents (e.g., silver or iodine), thereby endowing it with medicinal properties, their relatively rough surface texture and high absorbency cause them to become desiccated as exudate evaporates, which leads to adherence to the wound bed. Frequent removal of such adherent dressings can disrupt newly formed granulation tissue, resulting in secondary injury [66,67]. Furthermore, if not changed promptly, residual exudate that accumulates within the dressing provides a nutrient-rich medium that promotes bacterial proliferation and subsequent wound infection. Therefore, the suitability of traditional gauze and bandages for DWs depends on wound type, exudate level, infection status, dressing composition, and replacement frequency. In low-exudate, noninfected wounds that can be monitored and changed frequently, these simple conventional dressings may still serve a useful function.

However, for many chronic or highly exuding DWs, advanced dressings that maintain moisture balance and provide additional therapeutic properties are generally more appropriate. Currently, commonly used dressings include films and hydrogels, which can maintain the moisture level of the wound and facilitate its healing [68,69]. Among them, hydrogels have been widely studied due to their advantages of creating a closed space in the wound, keeping the wound moist, absorbing or managing wound exudate [70].

### Negative pressure wound therapy

Negative pressure wound therapy (NPWT) has gained prominence as an advanced adjunctive modality in the management of DWs [71]. The protocol typically involves initial surgical debridement followed by application of a sealed dressing connected to a vacuum source that delivers controlled subatmospheric pressure to the wound bed. Compared with standard dressings, NPWT enhances exudate clearance, reduces local concentrations of pro-inflammatory cytokines, improves tissue perfusion, and promotes granulation tissue formation [72]. Despite these advantages, NPWT does not circumvent the inherent limitations of conventional approaches. The prerequisite debridement step still poses infection risks, and the need for continuous device attachment often for weeks sunstantially restricts patient mobility and quality of life [73]. Consequently, long-term adherence remains suboptimal, limiting the real-world impact of this otherwise promising technology.

### Pressure off-loading and wound care

Mechanical stress represents a major, yet frequently underappreciated, impediment to healing in DWs [74]. Repetitive pressure and shear forces impair local perfusion, perpetuate tissue ischemia, and physically disrupt nascent granulation tissue [75]. Although this mechanical insult is most critical for plantar DFUs, pressure ulcers overlying bony prominences, including the sacrum, heels, and elbows, are also common in patients with diabetes and warrant comparable attention. Therefore, mechanical off-loading constitutes a cornerstone of DW management. For plantar DFUs, the gold standard is the total contact cast (TCC) or a nonremovable knee-high walker, both of which redistribute pressure across the entire foot and lower leg [76]. Removable walkers and half-shoes serve as alternatives; however, their efficacy is frequently compromised by poor patient adherence, as intermittent removal during ambulation negates the off-loading benefit [77]. For nonplantar wounds, off-loading can be achieved through specialized cushions, alternating-pressure mattresses, heel-relief boots, or, for bedridden patients, regular repositioning [78]. Custom insoles, therapeutic footwear, and felt padding are also employed to reduce focal pressure points. The International Working Group on the Diabetic Foot (IWGDF) guidelines recommend TCC or nonremovable walkers as first-line off-loading devices for plantar ulcers; for ulcers at other sites, a pragmatic approach combining pressure-redistribution devices with thorough patient education is essential [76]. Irrespective of the specific device, regular inspection of the skin beneath the off-loading apparatus and strict avoidance of direct pressure over the wound bed represent universally applicable principles.

### Vascular assessment and revascularization

Adequate arterial perfusion is an absolute prerequisite for wound healing at any anatomical site. In patients with diabetes, PAD is highly prevalent and frequently coexists with neuropathy, particularly in lower-extremity wounds; however, ischemic wounds can also occur at other sites as a consequence of systemic atherosclerotic disease [79]. Therefore, vascular assessment should be performed in all patients presenting with DWs at initial evaluation and repeated as clinically indicated. The ankle-brachial index serves as the standard screening tool, but may be falsely elevated (>1.3) in the presence of medial arterial calcification, a common feature in diabetes [80]. In such cases, the toe-brachial index, transcutaneous oxygen pressure (TcPO_2_), or skin perfusion pressure provides more reliable measures of distal perfusion [81]. TcPO_2_ values below 30 mmHg indicate critical ischemia and a poor healing potential, whereas values above 40 mmHg suggest sufficient perfusion to support wound closure [82]. Duplex ultrasound and magnetic resonance angiography (MRA) can further delineate anatomical stenoses or occlusions, and are particularly useful for characterizing lower-extremity wounds.

For patients with hemodynamically marked PAD, revascularization should be pursued without delay [83]. Endovascular interventions, including angioplasty and stenting, and surgical bypass, such as femoropopliteal or pedal bypass, constitute the primary revascularization modalities. The restoration of pulsatile blood flow markedly improves oxygen and nutrient delivery, enhances antibiotic penetration, and increases tissue viability. Importantly, revascularization should be performed before, or at the time of, major debridement or amputation to maximize the probability of healing. The same guiding principle applies irrespective of wound location: arterial insufficiency should be identified and corrected whenever feasible to optimize the wound bed for subsequent interventions.

### Nutritional support

Malnutrition constitutes a frequently overlooked comorbidity in patients with diabetes and chronic wounds. Protein-energy malnutrition impairs collagen synthesis, fibroblast proliferation, and immune function, all of which are essential for wound healing. Moreover, nutrient deficiencies of DW patients, including those of proteins, vitamins, and mineral salt, compromise antioxidant defenses and impair angiogenesis [84]. Therefore, nutritional assessment using validated screening tools, such as the Mini Nutritional Assessment or the Prognostic Nutritional Index, is recommended for all patients with nonhealing DWs. Dietary supplementation should provide adequate protein, energy and appropriate micronutrients, with intake guided by serum albumin, prealbumin, and trace element levels. In patients with severe malnutrition, oral nutritional supplements or enteral feeding may be necessary. Optimizing nutritional status not only accelerates wound closure but also reduces the risk of infection and improves overall clinical outcomes.

### Multidisciplinary collaboration and current clinical limitations

The multifactorial nature of DWs, encompassing metabolic dysregulation, vascular insufficiency, infection, mechanical stress, and nutritional deficits, necessitates a multidisciplinary team (MDT) approach. Optimal care integrates the expertise of endocrinologists, wound care specialists, vascular surgeons, infectious disease consultants, podiatrists, dietitians, and rehabilitation professionals [85]. Specialized DW clinics, in which these services are colocated, have consistently demonstrated important reductions in amputation rates and improved healing outcomes compared with fragmented, single-specialty care. Standardized care pathways, regular team conferences, and coordinated patient education constitute essential pillars of this collaborative model.

Despite these well-established benefits, the MDT approach, even when fully implemented, does not universally resolve the healing impasse in DWs, underscoring several profound and often intractable limitations. First, the effectiveness of the MDT is heavily resource-dependent and difficult to standardize across diverse healthcare settings. Comprehensive vascular assessment (e.g., TcPO_2_ and duplex ultrasound), timely revascularization, and access to advanced wound dressings or NPWT are frequently restricted to tertiary referral centers. In rural or under-resourced regions, patients commonly experience diagnostic delays, incomplete off-loading, and suboptimal glycemic titration, resulting in persistent disparities in clinical outcomes. Second, patient-related factors pose substantial barriers that even optimal multidisciplinary input cannot fully overcome. Strict adherence to off-loading regimens (e.g., continuous use of total contact casts), rigorous dietary modifications, and meticulous daily wound hygiene demand high levels of self-discipline and physical mobility. In elderly or frail patients with comorbidities such as visual impairment, neuropathy, or depression, adherence remains notoriously poor, directly undermining the efficacy of even the most sophisticated care protocols. Third, and most critically, current MDT interventions are fundamentally reactive and operate at a systemic or macroscopic tissue level, without directly addressing the recalcitrant molecular and cellular pathologies entrenched within the local wound microenvironment. Standard therapies, including debridement, systemic antibiotics, glycemic control, and pressure off-loading, do not actively eliminate persistent biofilms, neutralize excessive AGEs, relieve deep tissue hypoxia, or resolve the self-reinforcing cycles of oxidative stress and cellular senescence. Topical growth factors and conventional antimicrobials are subject to rapid enzymatic degradation, poor tissue penetration, and short half-lives, rendering them largely ineffective against biofilm-encased, metabolically dormant pathogens that perpetuate chronic inflammation. Consequently, even with ideal MDT execution, a substantial proportion of DWs remain stalled in the inflammatory phase, with recurrence rates reaching up to 40% within 1 year after healing and amputation rates remaining unacceptably high.

In essence, the MDT model provides an indispensable systemic scaffold for DW care, yet it lacks the tools to perform local, active microenvironmental reprogramming. This inherent gap between comprehensive systemic management and the need for intelligent, sustained, and multitargeted modulation of the wound bed precisely defines the niche where nanotechnology holds transformative promise. Nanomaterials, with their capacity for stimuli-responsive drug release, deep biofilm penetration, ROS scavenging, and spatiotemporal coordination of angiogenic and immunomodulatory signals, are uniquely positioned to complement and augment the MDT framework, addressing the biological obstacles that conventional multidisciplinary care cannot resolve.

## Nanomaterials for Remodeling the DW Microenvironment

The well-documented shortcomings of conventional DWs therapies highlight an urgent and unmet clinical need for innovative approaches capable of actively reprogramming the pathological wound milieu. Nanomaterials, by virtue of their tunable physicochemical properties, capacity for multifunctional integration, and ability to interface with biological systems at the relevant length scales, have emerged as a uniquely suited platform to address this challenge [86]. Central to their therapeutic potential is the capacity to normalize the dysregulated DWs microenvironment through coordinated, spatially and temporally controlled interventions that simultaneously target infection, oxidative stress, inflammation, and impaired tissue regeneration.

### The de novo design framework: From network deconstruction to temporal programming

Current nanomaterial-based strategies for DWs frequently present antibacterial, antioxidant, pro-angiogenic, and immunomodulatory functions as parallel therapeutic options, yet they rarely address 2 fundamental questions: which pathological node should be prioritized, and in what temporal sequence should distinct functions be deployed? In the absence of such a framework, even potent individual therapies can counteract one another, for instance, metal-based antimicrobials that generate ROS may exacerbate oxidative stress if administered before the wound’s redox burden is brought under control, thereby further compromising the microenvironment before healing can commence. To resolve this challenge, we propose a stratified, sequentially deployed logic that is grounded in the causal architecture of the DW pathological network.

Pathological factors in DWs can be stratified into 2 hierarchical layers. The primary drivers, sustained local hyperglycemia and resilient bacterial biofilms, function as autonomous upstream triggers [87]. Their persistence is independent of local inflammation, yet they continuously fuel downstream pathological cascades. The secondary responders, ROS overproduction, chronic inflammation, tissue hypoxia, and impaired angiogenesis, are host responses that are largely sustained by the ongoing activity of these primary drivers. A critical therapeutic implication follows from this hierarchical organization: upstream drivers must be neutralized first. If they remain active, downstream interventions, such as simple ROS scavenging or forced M2 macrophage polarization, will be transient at best and ultimately ineffective, because the initiating insults will continuously re-initiate the pathological cycle.

**Fig. 4.**
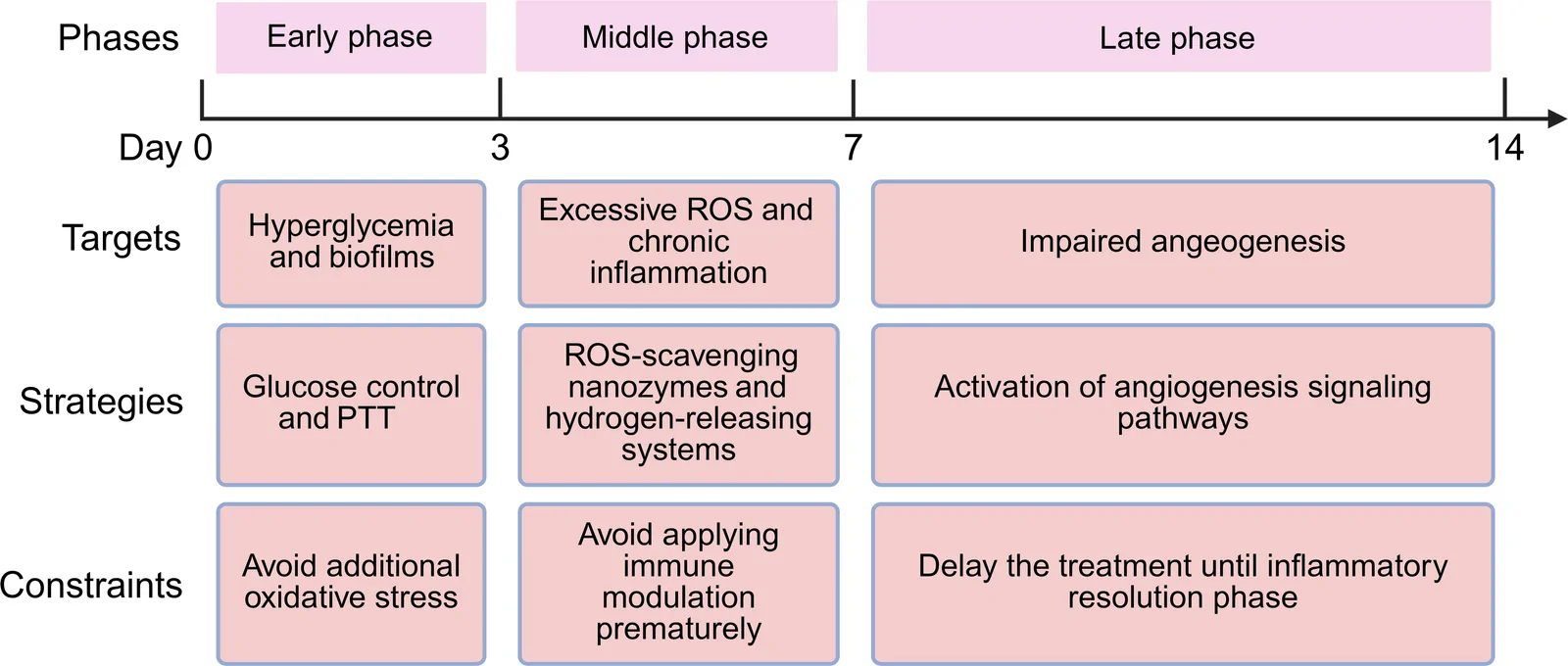
Temporally phased therapeutic roadmap for diabetic wounds. Diabetic wound pathological factors are stratified into upstream primary drivers and downstream secondary responders. Therapy should be administrated in sequential phases. In the early phase (days 0 to 3), local hyperglycemia and bacterial biofilms are prioritized to construct a healthy wound microenvironment without extra oxidative burden. In the middle phase (days 3 to 7), ROS elimination and inflammation resolution are carried out after infection control. In the late phase (days 7 to 14), pro-angiogenic strategies targeting the HIF-1α/VEGF axis are applied to generate mature blood vessels once inflammation fades. Premature implementation of downstream therapies will disrupt the healing cascade and impair therapeutic outcomes.

Based on this 2-tiered hierarchical architecture, we propose a temporally phased therapeutic roadmap (Fig. 4). In the early phase (days 0 to 3), intervention should concentrate on the primary drivers: local glucose levels must be reduced (e.g., through GOx- or probiotic-based systems) and resilient biofilms must be disrupted. Critically, biofilm clearance at this stage should preferentially employ strategies that do not impose an additional oxidative burden, such as mild photothermal heating (≤50 °C), rather than ROS-generating PDT, in order to establish a metabolically and microbiologically clean wound foundation without exacerbating oxidative injury. In the middle phase (days 3 to 7), once infection and hyperglycemia have been substantially mitigated, the focus shifts to the secondary responders. ROS-scavenging nanozymes or hydrogen-releasing systems can be deployed to neutralize the accumulated oxidative stress, and immunomodulatory interventions can be initiated to actively resolve inflammation. It is essential, however, that immune modulation not be applied prematurely, before the infectious burden is cleared, as untimely suppression of pro-inflammatory pathways may compromise host defense and impair bacterial clearance. In the late phase (days 7 to 14), when inflammation and oxidative stress have largely subsided, regenerative programs, particularly the HIF-1α/VEGF angiogenic axis, can be safely activated using extracellular vesicles (EVs) or growth factor-releasing carriers. Timing is decisive here: early VEGF stimulation, administered while inflammation is still active, tends to produce leaky, immature, and poorly perfused vessels that paradoxically worsen tissue edema and delay closure. Therefore, deferring pro-angiogenic therapy until a quiescent, noninflamed wound bed has been achieved is critical for generating functionally mature vasculature.

Notably, the ambitious objective of “reprogramming” the DW microenvironment necessitates a rigorous, quantifiable definition that transcends qualitative descriptions of therapeutic benefit. A successfully reprogrammed wound is not merely one in which an individual parameter is improved; rather, it is one in which the pathological network is demonstrably deconstructed and reconfigured toward a homeostatic set point. We propose that successful reprogramming should be defined by the concurrent achievement of several interconnected and quantifiable hallmarks, which can be systematically organized into 4 functional axes: restoration of redox homeostasis, immune homeostasis and resolution of inflammation, re-establishment of physiological function, and normalization of the biochemical milieu. Meanwhile, the reviewed nanotechnologies demonstrate this systems-level reprogramming not through any single “magic bullet” mechanism, but through their capacity to simultaneously and sequentially address multiple pathological nodes, an approach that aligns with our proposed 3-phase roadmap. Evidence for this system-level change is established when studies move beyond isolated endpoint measurements and provide a coordinated set of evidence: multiparameter analytics, temporal dynamics, bidirectional functional assays, and correlative histopathology. By evaluating nanomaterial strategies against this comprehensive, quantitative framework, the field can advance beyond the prevailing proof-of-concept paradigm and begin to rigorously assess whether a given platform genuinely achieves the ambitious goal of reprogramming the DW microenvironment. This systems-level, axis-based definition offers the conceptual clarity required to guide the rational design of next-generation nanomedicines, ensuring that therapeutic synergy is not merely proposed in theory but is demonstrably realized in practice.

In the following sections, each nanomaterial strategy classified by mechanism of action will be evaluated not merely on its intrinsic therapeutic efficacy, but also through the lens of its optimal temporal deployment window, its compatibility with interventions in adjacent phases, and the capacity of decoupling designs to spatially or temporally separate potentially conflicting functions, thereby averting counterproductive interactions. This framework marks a deliberate shift from the conventional paradigm of indiscriminate functional stacking where multiple activities are simply coloaded to an orchestrated synergy, in which every intervention is precisely timed to act on its designated pathological target. Such temporal coordination is essential to systematically dismantle the self-reinforcing vicious cycle that sustains the nonhealing state of DWs.

### Antibacterial strategies

The rampant overuse of broad-spectrum antibiotics has catalyzed the global rise of MDR bacterial pathogens, which not only jeopardize patient outcomes but also severely compromise the efficacy of standard wound care. In this context, nanotechnology-enabled antimicrobial strategies offer a compelling alternative by circumventing conventional resistance mechanisms. Guided by the hierarchical and temporal framework proposed previously, antibacterial strategies occupy a distinct, paramount position as the primary intervention during the early phase (days 0 to 3). Biofilms, as upstream primary drivers, autonomously fuel the downstream oxidative and inflammatory cascades; their persistence renders later interventions futile. Consequently, unlike anti-inflammatory or pro-angiogenic therapies, which are optimally deployed in later phases, antibacterial nanotherapies must be initiated at the very onset of treatment to dismantle the pathological network at its root. Their primary objective is not merely to reduce the bacterial burden, but to irreversibly disrupt the biofilm state, thereby establishing a sterile, metabolically quiescent wound foundation upon which subsequent healing phases can proceed. A critical consideration during this phase is the avoidance of strategies that impose an additional oxidative burden, especially uncontrolled ROS generation, as this would exacerbate the already compromised redox state of the wound bed. Instead, early-stage antibacterial interventions should prioritize biofilm disruption and bacterial eradication with minimal collateral oxidative injury, a requirement that directly shapes the selection and design of appropriate nanotechnologies.

Beyond conventional antibiotics, PTT and PDT have emerged as nonantibiotic, nanomaterial-based strategies for combating biofilm-encased pathogens in DWs [88]. Although both modalities rely on external light irradiation, they operate through fundamentally distinct physical mechanisms: PTT converts light energy into localized hyperthermia to thermally ablate bacteria, whereas PDT generates cytotoxic reactive species via photosensitizer-mediated energy transfer. A shared challenge, however, lies in balancing potent antimicrobial efficacy against collateral injury to surrounding host tissues, a trade-off that has motivated recent innovations in the precise regulation of thermal dose and the spatiotemporal control of reactive species.

**Fig. 5.**
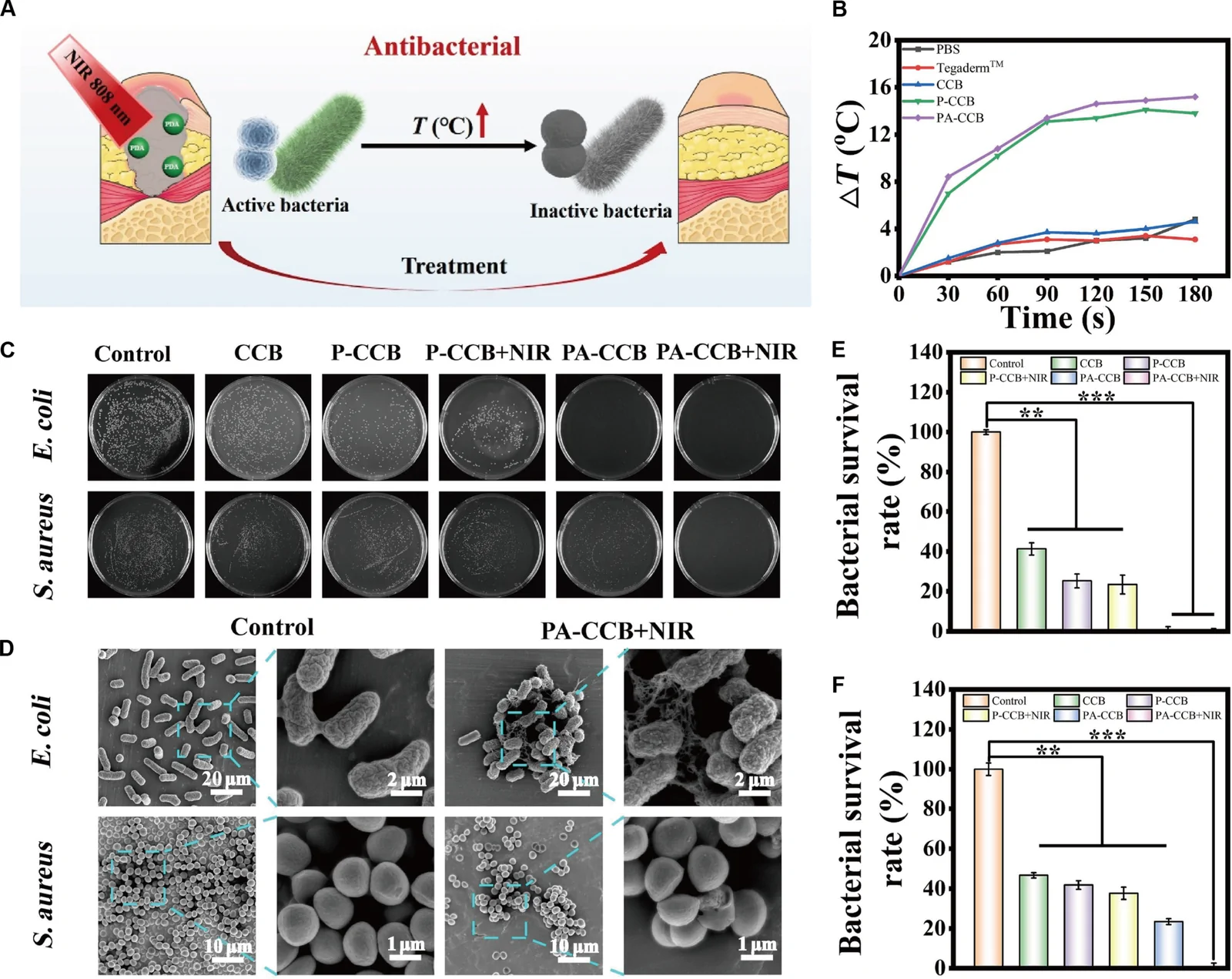
Antibacterial effect of PTT. (A) Under the treatment of NIR, homologous nanomaterials produce high temperature, thereby causing death of bacteria. Reproduced with permission [262]. Copyright 2022, Elsevier. (B) Photothermal effect of Ag@MPDA nanoparticles, with the temperature approximately 50 °C. Reproduced with permission [90]. Copyright 2025, Elsevier. (C to F) Ag@MPDA nanoparticles have robust antibacterial effects toward *E. coli* (99.9%) (E) and *S. aureus* (99.7%) (F). Reproduced with permission[90]. Copyright 2025, Elsevier.

PTT employs photothermal agents to induce rapid local temperature elevation. Early designs, such as the Fe_3_O_4_@MoS_2_ core–shell nanozyme, achieve impressive killing of methicillin-resistant *S. aureus* (MRSA) (~98.6%) under near-infrared (NIR) irradiation. However, the localized temperature reaches approximately 60 °C, causing irreversible injury to peri-wound fibroblasts and keratinocytes, while the concomitant chemodynamic generation of ROS exacerbates oxidative stress in the already compromised diabetic microenvironment [89]. To overcome these dual drawbacks, He et al. [90] design a chitosan-guar gum hydrogel incorporating Ag@mesoporous polydopamine (Ag@MPDA) nanoparticles. The hydrogel matrix functions as a thermal insulator that self-limits the photothermal temperature to below 50 °C through localized surface plasmon resonance, achieving 99.7% and 99.9% killing of *S. aureus* and *Escherichia coli*, respectively, without thermal damage to host tissue (Fig. 5). Critically, the polydopamine component provides intrinsic antioxidant activity, scavenging excess ROS within the wound bed. This design therefore reconciles effective bacterial ablation with cytoprotection, representing a paradigm shift from purely aggressive photothermal ablation to microenvironment-responsive, self-regulated thermal therapy.

**Fig. 6.**
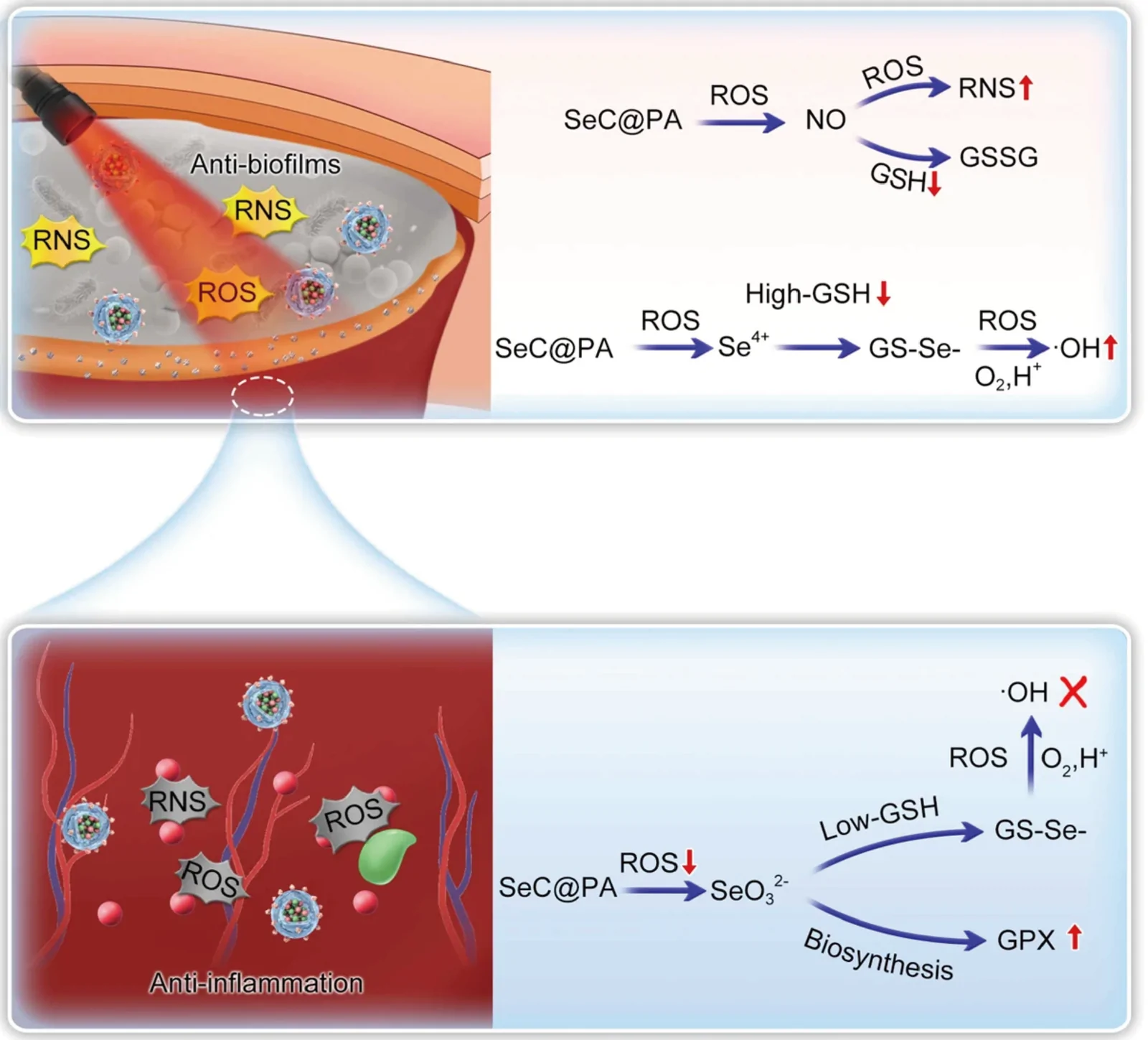
Sec@PA MN has robust PDT activity while bypassing injuries toward normal tissue caused by ROS and RNS. Reproduced with permission [10]. Copyright 2023, Springer Nature.

PDT generates ROS and other reactive species through the activation of photosensitizers, but the indiscriminate oxidative burst poses comparable risks to surrounding host tissue [91]. To address this challenge, Yang et al. [10] developed a bioresponsive microneedle (MN) patch (SeC@PA MN) that achieves spatially selective regulation of reactive species by exploiting the endogenous glutathione (GSH) gradient that is naturally present across DW compartments. Biochemical quantification confirms that biofilm-laden wound coverings contain approximately 9.3-fold higher GSH (6.69 mmol/g) than the underlying viable tissue (0.72 mmol/g). Within the high-GSH biofilm layer, the SeC@PA system not only depletes excess GSH to block reactive species quenching but also triggers a cascade under 660-nm laser: photooxidation of L-arginine to nitric oxide (NO) and subsequent formation of reactive nitrogen species (RNS), together with selenium oxidation to generate hydroxyl radicals (•OH). The cascade collectively produces a localized “RS storm” capable of thoroughly eradicating entrenched bacteria. In contrast, within the low-GSH viable tissue beneath the biofilm, the same platform switches to potent RS-scavenging activity, neutralizing PDT-derived radicals and up-regulating GPX4 to alleviate oxidative stress. The dissolvable MN patch physically penetrates the biofilm and exudate barriers, simultaneously delivering nanoparticles to both compartments and thereby enabling dual-directional RS modulation: pro-oxidative in the infected surface layer and anti-oxidative in the regenerative parenchyma (Fig. 6). This spatial selectivity, validated by ex vivo and in vivo fluorescence mapping and electron paramagnetic resonance (EPR) detection, provides a blueprint for precision PDT that circumvents the indiscriminate oxidative injury typical of conventional photosensitizers. Nevertheless, the clinical translatability of this GSH-gradient-dependent strategy requires further validation in human diabetic ulcers, where polymicrobial biofilms and heterogeneous GSH distribution may attenuate the sharp gradient observed in murine models.

Despite the sophistication of spatially selective PDT strategies, their clinical translation for DWs is constrained by several inherent physical and biological limitations. First, PDT is critically oxygen-dependent, as type II photochemical reactions consume molecular oxygen to generate cytotoxic singlet oxygen; however, DWs are profoundly hypoxic due to microvascular dysfunction, creating a self-limiting paradox in which the antimicrobial modality is progressively attenuated by the very microenvironment it is intended to treat [92]. Second, light penetration is limited to a few millimeters even within the NIR window, which is insufficient for deep biofilms or thickened hyperkeratotic margins [93]. Moreover, the irregular, 3-dimensional topography of ulcers, combined with exudate-induced scattering, prevents uniform fluence delivery, leading to underdosed regions that permit pathogen persistence and overdosed zones that injure host tissue. Third, most critically, despite advanced designs that exploit GSH gradients to spatially confine reactive species, most PDT platforms lack such selectivity, and photogenerated ROS indiscriminately damage senescent fibroblasts and keratinocytes, thereby exacerbating oxidative stress and delaying re-epithelialization, which makes PDT pose a fundamental paradox for early-phase (days 0 to 3) application [92]. Even with spatial control, the unavoidable production of reactive species conflicts with the primary objective of minimizing additional oxidative injury in a wound bed already overwhelmed by oxidative stress. The risk of collateral damage to senescent fibroblasts and keratinocytes in the peri-wound tissue remains high, potentially delaying subsequent re-epithelialization. Therefore, despite its elegant design, we argue that PDT-based strategies, particularly those that generate high ROS fluxes, should be deferred or carefully combined with potent, immediate ROS-scavenging systems to prevent them from counteracting the goal of establishing a noninflammatory foundation for later-stage therapies. Collectively, these constraints, including oxygen dependence, limited penetration depth, irradiation nonuniformity, and host phototoxicity, underscore that PDT requires careful contextual application. Future studies must validate its efficacy in clinically relevant hypoxic, polymicrobial, and irregular wound geometries, and consider combination with oxygen-replenishing systems or alternative modalities to offset these shortcomings.

Moreover, regarding the translatability of this spatially selective response within heterogeneous human DWs, we acknowledge inherent clinical variability as a key limitation requiring further investigation. Human DFUs feature polymicrobial biofilm aggregates with uneven GSH distribution, which may lead to the failure of the sharp GSH gradient observed in standardized murine wound models [94]. Therefore, future translational work will optimize MN loading density and laser irradiation protocols to accommodate variable biofilm thickness, and validate bidirectional reactive species regulation under clinically relevant polymicrobial infection conditions to confirm consistent spatial therapeutic performance in complex human wound microenvironments.

Beyond PTT and PDT, metal-based nanomaterials represent a prominent class of antibacterial agents for DWs management. Their antimicrobial efficacy primarily derives from the controlled release of metal ions, such as Ag^+^, Zn^2+^, and Cu^2+^, which act through pleiotropic mechanisms to compromise bacterial viability (Table 1) [95–97]. These include membrane destabilization, induction of oxidative stress, and interference with essential enzymatic and genetic processes, collectively culminating in robust bactericidal activity. Each metal-based nanomaterial is investigated with distinct physicochemical properties, antibacterial spectra, and safety profiles.

**Table 1.** Size, morphology, and species-dependent antibacterial mechanisms of metal-based nanomaterials and their associated cytotoxicity profiles

Type	Mechanisms	Size	Morphology dependency	Antibacterial spectrum	Toxicity concentration	Ref.
AgNPs	1. Membrane disruption 2. Intracellular oxidative stress 3. Inhibiting transcription and translation	≤10 nm: Robust antibacterial activity	Triangular nanoplates release Ag^+^ faster than spherical or cubic counterparts	Broad-spectrum, especially gram-negative bacteria	Fibroblasts: 14.98 μg/ml	[100–102]
Zn-MOFs	1. Membrane disruption 2. Intracellular oxidative stress 3. Enzymatic interference	40–200 nm	Depending on porosity and ligand type	Broad-spectrum	Depending on size, morphology, and ligands	[105]
CuNPs	1. Membrane disruption 2. Intracellular oxidative stress 3. Aberrant filamentation	40–80 nm	Porous structure effect is more pronounced	Broad-spectrum	0.001–100 μg/ml (to RAW 264.7)	[108,264]
CuONPs	1. Membrane and cell wall disruption 2. Intracellular oxidative stress	≤100 nm	Spherical and rod-shaped nanoparticles show effective antibacterial activity.	Broad-spectrum	122.5 ppm (to HEK293T cells)	[265,266]
Cu-MOFs	1. Membrane disruption 2. Intracellular oxidative stress	100–200 nm	Depending on porosity and ligand type	Broad-spectrum	2.0 μg/ml (to embryos of the marine zebrafish)	[267]

Silver-based nanomaterials, particularly silver nanoparticles (AgNPs), represent the most clinically advanced class of antimicrobial wound dressings, with several Food and Drug Administration (FDA)-approved products (e.g., Silvercel, SilverStat, and SilverSorb) already in routine use [98]. Their antibacterial activity is mediated by the sustained release of Ag^+^ ions, which bind to negatively charged bacterial membranes, increase membrane permeability, and induce cytoplasmic leakage [99]. Once internalized, Ag^+^ catalyzes the generation of ROS and inhibits both DNA replication and ribosomal function, thereby halting protein synthesis. The antibacterial potency and cytotoxicity of AgNPs are strongly size- and shape-dependent: particles smaller than 10 nm exhibit robust activity, whereas triangular nanoplates release Ag^+^ more rapidly than their spherical or cubic counterparts [100,101]. However, the therapeutic window for AgNPs is notably narrow. At concentrations below 2 μg/ml, their activity against multidrug-resistant (MDR) pathogens is limited, while doses exceeding 10 μg/ml cause substantial cytotoxicity to primary human dermal fibroblasts and keratinocytes [102]. Following systemic absorption, Ag^+^ accumulates predominantly in several organs including liver and kidneys, with a terminal half-life of weeks to months, raising concerns regarding chronic argyria and hepatorenal toxicity.

Zinc-based systems, particularly Zn-MOFs such as ZIF-8 and ZIF-90, represent a biocompatible alternative, given the essential role of zinc in cellular metabolism [103]. Their pH-responsive degradation enables spatiotemporally controlled release of Zn^2+^ within the acidic wound microenvironment [104]. The liberated Zn^2+^ exerts antibacterial effects through membrane disruption, the generation of ROS, and competitive displacement of native metalloenzyme cofactors, for example, Mg^2+^, leading to metabolic collapse [105]. Importantly, the biological response to Zn^2+^ is strongly concentration-dependent: while concentrations below 50 μM promote fibroblast proliferation, levels exceeding 200 μM induce oxidative stress and apoptosis via the JNK/p38 MAPK pathways [106]. Furthermore, the organic linkers, such as imidazole derivatives, may exert independent cytotoxic or immunogenic effects; however, their metabolic fate remains poorly characterized [107]. Systemic absorption of topically applied Zn^2+^ is generally low (less than 5% of the applied dose) through intact skin, but it increases substantially in ulcerated wounds, underscoring the necessity for wound type-specific safety evaluations.

Copper-based nanomaterials, including copper nanoparticles (CuNPs), copper oxide nanoparticles (CuONPs), and Cu-MOFs, are highly potent bactericides but also present the most grave safety challenges. CuNPs induce aberrant bacterial filamentation through failed septation and disrupt cell wall integrity, whereas CuONPs inflict direct physical damage to bacterial membranes [108,109]. Cu-MOFs exploit the acidic pH of the wound microenvironment to release Cu^2+^ ions, which act as potent Fenton catalysts, generating hydroxyl radicals (·OH)[110]. Critically, this burst of ROS damages both bacterial and host cells: in human dermal fibroblasts, Cu^2+^ concentrations as low as 10 to 25 μM compromise mitochondrial membrane potential and deplete intracellular GSH, while bactericidal concentrations markedly inhibit keratinocyte proliferation [111]. In vivo, high-dose CuNP application is associated with localized necrosis and delayed granulation tissue formation, although sustained low-dose release from Cu-MOFs exhibits a more favorable therapeutic index [112]. Given that diabetic tissues already display elevated copper levels owing to impaired ceruloplasmin function, exogenous supplementation may further disturb systemic homeostasis, and chronic exposure has been linked to hepatotoxicity and neurotoxicity in preclinical models.

While the ROS-generating capacity of metal-based nanoparticles underpins their potent bactericidal activity, a critical consideration is their selectivity toward prokaryotic cells over eukaryotic cells within the wounded milieu. The selectivity stems from several biophysical distinctions: (a) the smaller size of bacteria provides a higher surface-area-to-volume ratio for nanoparticle interaction; (b) the absence of a complex endocytic machinery in bacteria allows for direct membrane penetration, whereas mammalian cells primarily internalize nanoparticles via slower, energy-dependent endocytosis; and (c) mammalian cells possess more robust antioxidative defense systems (e.g., higher basal GSH levels) compared to bacteria. Nevertheless, this selectivity is highly dose- and surface-modification-dependent [113–115]. Excessive concentrations of Ag^+^, Zn^2+^, or Cu^2+^ can overwhelm the antioxidative capacity of keratinocytes, fibroblasts, and endothelial cells, inducing apoptosis and impairing re-epithelialization. To mitigate this, recent strategies have focused on engineering “selective nano-antibiotics”, such as coating AgNPs with mammalian-cell-repelling zwitterionic polymers or utilizing bacteria-specific pH/ATP-triggered release mechanisms, ensuring that high antimicrobial doses are achieved only in the immediate vicinity of the pathogen. For instance, Xiang et al. [116] developed a mussel-inspired poly(sulfobetaine methacrylate-co-dopamine methacrylamide) copolymer that both reduces AgNO_3_ to AgNPs and anchors the zwitterionic polymer onto the nanoparticle surface in a one-step synthesis. The resulting zwitterionic AgNPs, incorporated into an injectable gelatin methacryloyl/poly(vinyl alcohol) hydrogel, demonstrated substantially improved antibacterial efficiency compared to non-zwitterionic counterparts both in vitro and in vivo, while the zwitterionic modification also provided enhanced hemocompatibility and biocompatibility. Ding et al. [117] constructed a multifunctional nanospray (MXene/ZIF-90@ICG) by incorporating ZIF-90@ICG nanoparticles onto MXene-NH_2_ nanosheets. This system achieves on-demand release of antimicrobial factors including MXenes, ICG, and Zn^2+^ in response to variations in both pH and ATP levels within the bacterial infection microenvironment. Collectively, these representative examples demonstrate that rational design of surface chemistry and stimuli-responsive release mechanisms can effectively confine antimicrobial activity to the pathogen-rich wound microenvironment, thereby achieving a favorable balance between potent antibacterial efficacy and preservation of host tissue integrity, which is a critical consideration for the clinical translation of nanomaterial-based wound therapeutics.

Moreover, emerging evidence suggests that bacteria are not defenseless against metal ion stress. Prolonged sub-inhibitory exposure to Ag^+^ or Cu^2+^ has been shown to up-regulate efflux pump systems (e.g., the cus and sil operons in *E. coli*), thereby increasing the intracellular export of toxic ions [118]. The oxidative stress induced by nanoparticles can act as a signal for biofilm matrix overproduction, leading to a phenotype of enhanced tolerance rather than genetic resistance [119]. This raises a subtle but critical concern: the nonspecific stress response may inadvertently promote a biofilm-rich, tolerant state that complicates wound management. To counteract this adaptation, current nanomedicine design advocates for “combination synergy” rather than monotherapy. Codelivery of metal ions with conventional antibiotics or PTT creates a simultaneous triple assault (ROS + drug + heat), which drastically lowers the minimum inhibitory concentration and reduces the window for adaptive evolution [120].

A unique advantage of metal-organic framework (MOF) platforms lies in their highly ordered porous architecture, which enables codelivery of auxiliary therapeutics (e.g., antibiotics or growth factors) alongside metal ions [121]. This “metal ion + drug” synergistic strategy not only amplifies antimicrobial potency but also mitigates the selective pressure driving antibiotic resistance. However, the translational potential of all metal-based nanosystems is constrained by several cross-cutting issues. First, most cytotoxicity data derive from short-term (24 to 72 h) in vitro assays using immortalized cell lines, which poorly predict chronic, low-dose exposure in clinical wound care. Second, the effects of metal ions on senescent cells that are abundant in DWs remain largely unexplored; senescent fibroblasts exhibit altered metal uptake and oxidative sensitivity, potentially narrowing the therapeutic window. Third, standardized methodologies for evaluating ion-release kinetics, degradation rates, and tissue accumulation across different wound exudate conditions are lacking, hampering interstudy comparisons and regulatory decision-making. Fourth, the interplay between sublethal metal-ion concentrations and bacterial virulence, biofilm architecture, and horizontal gene transfer remains inadequately understood.

To resolve the inherent conflict between potent antimicrobial activity and collateral oxidative injury to host cells, rational design principles must incorporate the temporal decoupling strategies outlined in the “The de novo design framework: from network deconstruction to temporal programming” section. Uncontrolled ion release can provoke a sudden burst of ROS that exacerbates tissue damage before healing can commence. This challenge can be addressed through several complementary design approaches: (a) antioxidant surface coatings (e.g., polydopamine and ceria shells) that scavenge free radicals in situ, thereby enabling concurrent bacterial eradication and cytoprotection; (b) stimuli-responsive encapsulation within pH or enzyme-labile matrices, which delays ion release until the carrier reaches the acidic biofilm microenvironment, preventing early toxic peaks and allowing endogenous defense systems to adapt; and (c) core–shell architectures in which the metal core is positioned beneath an enzyme-active outer layer, ensuring that the antibacterial payload is liberated only after preceding therapeutic tasks, for example, glucose depletion and ROS scavenging, have been completed. Collectively, these design principles transform the ion-ROS conflict from an inherent liability into a programmable, sequential synergy, guaranteeing that metal-based antimicrobials are deployed only after the local redox milieu has been stabilized, thereby minimizing collateral injury while preserving potent bactericidal efficacy. Future studies should therefore prioritize long-term (≥4 weeks) biocompatibility assessments in relevant diabetic animal models, including histological examination of peri-wound tissues, quantification of metal deposition in regional lymph nodes and distant organs, and evaluation of effects on wound biomechanics and scar quality, alongside the development of ion-chelating “safety switches” that enable rapid neutralization should local toxicity occur.

In summary, effective antibacterial therapy constitutes the indispensable first step (days 0 to 3) in the temporally phased therapeutic roadmap. Well-designed nanomaterials, such as those employing mild PTT or ion-releasing systems with antioxidant coatings, enable the critical early-phase milestone of complete biofilm disruption and pathogen eradication without superimposing grave oxidative injury. Successfully accomplishing this task establishes the foundation for the middle-phase interventions (days 3 to 7), which focus on scavenging accumulated ROS and resolving the dysregulated immune response. Only after a clean, infection-free, and metabolically stable wound bed has been established can late-phase pro-angiogenic strategies be safely and effectively deployed. Thus, from a systems perspective, the antibacterial phase is not an isolated function but rather the foundational layer upon which the entire therapeutic sequence is built.

### Blood glucose control and regulation of oxidative stress response

Hyperglycemia constitutes the principal pathological driver of impaired healing in DWs [122]. Consequently, a major thrust in therapeutic development has focused on strategies that enable localized glucose reduction within the wound microenvironment. Concurrently, oxidative stress, long recognized as a key coconspirator in healing dysfunction, has prompted the integration of potent antioxidant activity into contemporary nanotherapeutic designs [123,124]. This dual-targeting paradigm simultaneously addresses 2 interlinked pathophysiological hallmarks, thereby fostering a more conducive milieu for tissue repair [103]. The following section highlights representative nanosystems engineered to fulfill this dual-functional role.

**Fig. 7.**
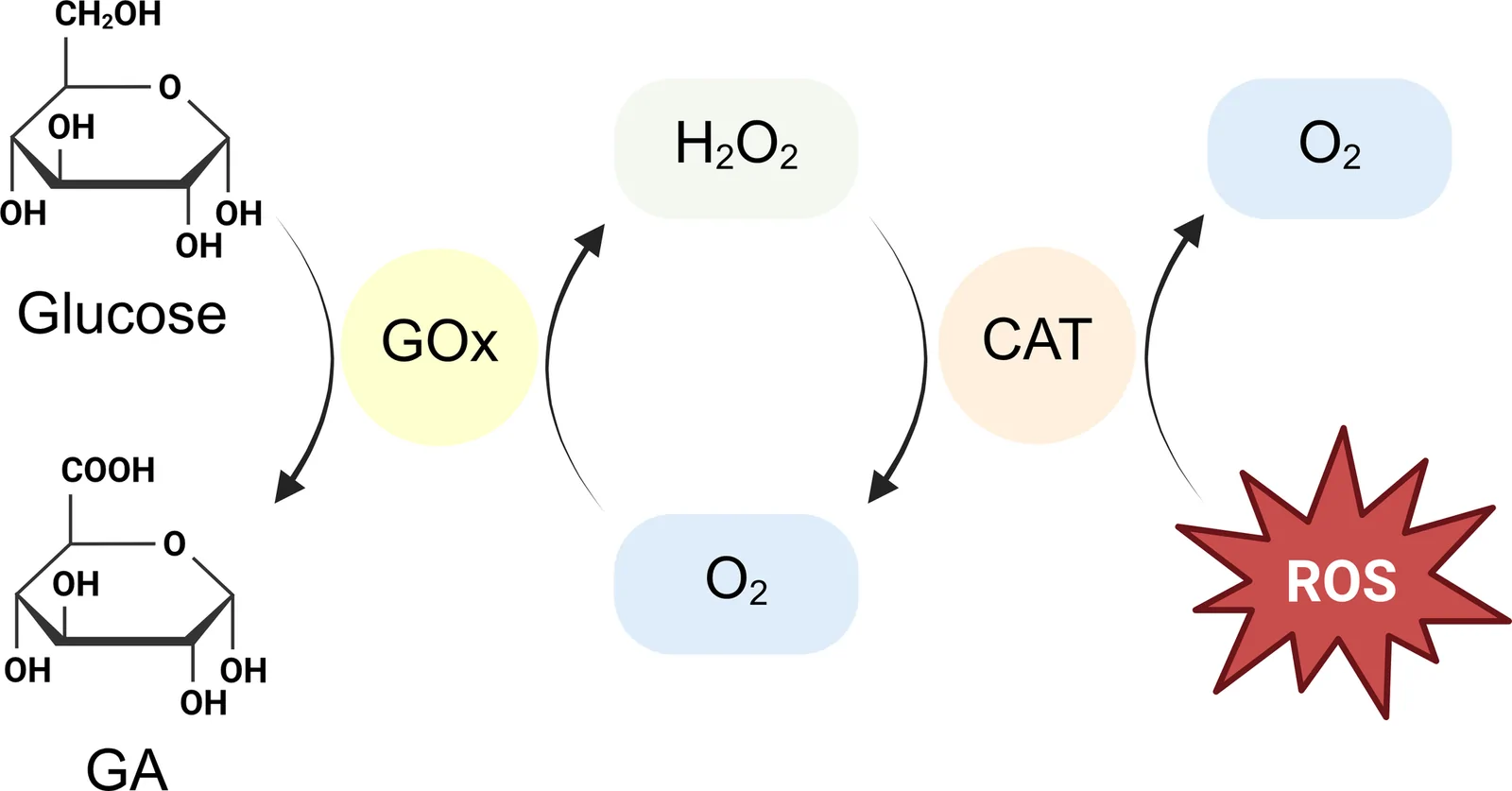
The mechanism of the GOx-based system in treating DWs. Glucose is decomposed into GA and H_2_O_2_ under the catalysis of GOx. H_2_O_2_ is decomposed by CAT to produce oxygen, which can supply oxygen to hypoxic tissues at the wound site.

GOx catalyzes the oxidation of glucose to gluconic acid and hydrogen peroxide (H_2_O_2_), thereby lowering local glucose concentrations while simultaneously generating a mildly acidic environment and a ROS by-product [125]. These outputs are not merely metabolic waste but can be strategically harnessed as functional triggers: the acidification can be exploited to disassemble pH-labile carriers, including MOFs, enabling spatiotemporally controlled drug release, whereas the H_2_O_2_, if subsequently decomposed by catalase (CAT) or CAT-mimetic nanozymes, can provide a local oxygen supply to alleviate tissue hypoxia (Fig. 7). However, the oxygen balance in GOx/CAT cascade systems warrants careful quantitative consideration. Stoichiometrically, the GOx-catalyzed oxidation of 1 mol of glucose consumes 1 mol of O_2_ to generate 1 mol of H_2_O_2_, whereas the subsequent decomposition of this H_2_O_2_ by CAT regenerates only 0.5 mol of O_2_. Consequently, the GOx/CAT cascade inherently results in a net consumption of 0.5 mol of O_2_ per mole of glucose, which, if uncontrolled, risks exacerbating local hypoxia in severely ischemic wound beds. A net re-oxygenation effect can be achieved only under specific formulation and microenvironmental conditions: when CAT or CAT-mimicking nanozymes also decompose the abundant endogenous H_2_O_2_ and other ROS that have accumulated in the pathological DW, thereby supplying supplementary O_2_ beyond that derived from the GOx pathway; or when the nanosystem incorporates codelivered oxygen carriers or oxygen-generating agents, such as perfluorocarbon emulsions or inorganic peroxides. Therefore, optimizing the catalytic kinetic ratio between GOx and CAT/nanozymes and tailoring formulation parameters to local oxygen availability are essential to ensure that glycemic control does not compromise tissue oxygenation [126].

Moreover, this enzymatic cascade carries an inherent paradox: the unavoidable coproduction of H_2_O_2_ exacerbates oxidative stress in the absence of immediate scavenging mechanisms, and excessive GOx may lead to over-acidification or oxygen depletion, both of which are detrimental to wound healing. Moreover, the dynamic range of current GOx nanoreactors, typically beginning within 5 to 15 min and responsive to 10 to 40 mM glucose, is calibrated against uniform buffered solutions, whereas wound interstitial fluid glucose exhibits delayed, dampened peaks and pronounced spatial gradients that are rarely recapitulated in preclinical models, leaving a critical translational gap.

To harness the acid-triggering capability of the GOx cascade while mitigating its oxidative liability, several cascaded nanoplatforms have been engineered. Wei et al. [127] construct a GOx@ZIF-90-Arg nanoreactor embedded in a hydrogel, coencapsulating GOx and L-arginine (L-Arg) within a zeolitic imidazolate framework-90 (ZIF-90). In the hyperglycemic wound milieu, GOx catalyzes glucose oxidation, generating gluconic acid and H_2_O_2_; the resultant pH drop triggers disassembly of ZIF-90, releasing Zn^2+^ ions that confer broad-spectrum antibacterial activity and L-Arg. Simultaneously, H_2_O_2_ oxidizes L-Arg to NO, a potent angiogenic signal. This cascade concurrently lowers local glucose, combats infection, and promotes neovascularization. However, its catalytic output is highly substrate-dependent: the yields of H_2_O_2_ and NO fluctuate with wound glucose concentrations, leading to variable therapeutic intensity across patients. Moreover, the one-pot biomineralization synthesis suffers from batch-to-batch variability, sustained Zn^2+^ leakage poses cytotoxic risks to regenerative cells, and the porous carrier readily adsorbs proteins from wound exudate, accelerating premature degradation.

Similarly, Jiang et al. [128] develop an activatable FCSGP nanoreactor that disintegrates in the acidic DW microenvironment, liberating Fe^2+^ and Cu^2+^ ions for synergistic antibacterial and Fenton-like oxidative effects, while also functioning as a photothermal transducer under NIR irradiation. The mild hyperthermia not only enhances bacterial killing but also boosts GOx activity, creating a positive feedback loop that amplifies glucose consumption and acidification. Yet, this loop is tightly coupled to substrate availability and local buffering capacity; highly exuding wounds may attenuate acidification, impairing ion release, and the long-term toxicity of transition metal ions, particularly if they escape the wound site, remains insufficiently characterized. Furthermore, the complexity of integrating multiple metal sources, an enzyme, and a photothermal agent into a single construct complicates manufacturing scale-up and quality control.

Despite the promising efficacy of GOx-based nanoreactors in preclinical models, their clinical translation demands rigorous evaluation of the desirability and controllability of local glucose depletion. From a clinical standpoint, the therapeutic window is delicately poised: while moderate glucose reduction ameliorates hyperglycemia-driven dysfunction, excessive GOx activity can inadvertently trigger a cascade of detrimental events within the ischemic wound bed. First, the excessive production of gluconic acid can overwhelm local buffering capacity, driving the pH below the physiological threshold, which not only inhibits fibroblast proliferation and collagen deposition but also activates acid-sensing nociceptors, causing patient discomfort [129]. Second, even when CAT or CAT-mimetic nanozymes are coincorporated to decompose H_2_O_2_ into O_2_, an unregulated GOx loading density can transiently deplete local O_2_ faster than it is replenished, which is a phenomenon termed the “oxygen paradox”, thereby exacerbating the preexisting hypoxia characteristic of DWs [130]. Third, the unavoidable leakage or burst release of H_2_O_2_ during the initial catalytic phase, if exceeding the scavenging capacity of the coformulated antioxidant modules, inflicts direct oxidative injury on peri-wound keratinocytes and vascular endothelial cells (VECs), ultimately offsetting the pro-healing benefits [131].

To render this strategy clinically viable, future designs must incorporate explicit response thresholds and safety cutoffs. Ideally, intelligent GOx nanoplatforms should be engineered with glucose concentration-dependent ON–OFF switches that ensure that the catalytic cascade is robustly activated only under pathological hyperglycemic conditions (e.g., local glucose > 400 mg/dl) and spontaneously attenuates as glucose declines toward normoglycemic levels (<100 mg/dl)[132]. Concurrently, the enzymatic payload per nanocarrier must be optimized to maintain a local pH of 6.5 to 7.4 and an oxygen tension above 30 mmHg, thereby achieving a clinically acceptable balance between antibacterial and glucose-lowering efficacy on the one hand and host tissue preservation on the other. The incorporation of highly efficient multienzyme cascades, such as GOx-CAT or GOx-peroxidase nanozymes, with precisely tuned molar ratios represents a promising strategy to establish this fail-safe mechanism, ensuring that the rate of H_2_O_2_ generation is tightly coupled to its rapid neutralization [133].

To circumvent the injury caused by H_2_O_2_, Wang et al. [134] design a probiotic-active hydrogel (LR&AB@CAH) that integrates the lactic acid bacterium *Lactobacillus reuteri* (LR) with acid-responsive hydrogen-generating nanoparticles. In this system, the living probiotic metabolizes local glucose to produce lactic acid, thereby lowering the wound pH; this acidic microenvironment subsequently triggers the nanoparticles to release molecular hydrogen (H_2_), a selective ROS scavenger that neutralizes oxidative stress without generating cytotoxic H_2_O_2_. This design fundamentally rewires the metabolic output from an oxidative to a reductive profile, while the mild acidification concurrently suppresses the growth of pathogenic bacteria. The calcium alginate matrix preserves bacterial viability and provides mechanical stability.

However, it is important to avoid characterizing lactic acid as intrinsically “physiologically benign”, as its biological effects are strongly concentration-dependent. Although lactate has the function of promoting M2 macrophage polarization, in the DW microenvironment, excessive local lactate accumulation (e.g., ≥ 4 to 5 mM) and the accompanying reduction in pH can impair ECM remodeling and exacerbate fibroblast dysfunction [135–137]. Furthermore, the clinical translation of this probiotic-based platform faces formidable hurdles that extend well beyond its catalytic efficacy. The viability and metabolic activity of *L. reuteri* are highly sensitive to storage temperature, oxygen exposure, and formulation shear stress, raising substantial concerns about long-term shelf life and batch-to-batch reproducibility [138,139]. The microbe-associated molecular patterns present on the bacterial surface, such as LTA and peptidoglycan, can be recognized by host pattern-recognition receptors, notably toll-like receptors, on wound-resident macrophages [140]. Without precise characterization of the strain’s immunomodulatory profile, such recognition may inadvertently activate pro-inflammatory signaling cascades, thereby paradoxically exacerbating local inflammation. More critically, the introduction of live bacteria into open, necrotic, and immunocompromised diabetic ulcers carries an inherent risk of opportunistic infection, demanding rigorous biosafety validation [141]. From a regulatory standpoint, such living therapeutic agents are subject to far more complex oversight than conventional nanomaterials or enzymes, requiring distinct frameworks for quality control, sterilization without loss of bacterial viability, and comprehensive in vivo biodistribution and persistence studies. Therefore, although the GOx-free probiotic strategy provides an elegant biochemical workaround for H_2_O_2_-mediated toxicity, its ultimate therapeutic potential hinges on effectively resolving these intertwined challenges: local metabolic impact, long-term storage stability, infection liability, and regulatory pathfinding.

Collectively, these designs delineate a progressive evolution from simple glucose-consuming enzymes to integrated cascades that repurpose reaction by-products, including acid, H_2_O_2_, or NO, for secondary therapeutic benefits, and further to nonoxidative, living systems that circumvent ROS generation entirely. Importantly, the therapeutic success of these glucose-modulating platforms hinges critically on their temporal deployment within the wound healing cascade.

Guided by the 3-phase roadmap proposed previously, local glucose reduction should be initiated during the early phase (days 0 to 3), as sustained hyperglycemia acts as an upstream primary driver that continuously fuels downstream oxidative and inflammatory cascades. However, the choice of technology during this phase demands caution: conventional GOx-based systems, although effective in lowering glucose, simultaneously generate H_2_O_2_, which can exacerbate oxidative injury in a wound bed that has not yet been relieved of its initial redox burden and where endogenous antioxidant defenses are already overwhelmed. Therefore, early-phase application is better served by nonoxidative alternatives, such as probiotic-based lactate-producing systems or hydrogen-releasing platforms that achieve glucose depletion without superimposing additional oxidative insult. In parallel, biofilm eradication, another primary driver, must either precede or be coordinated with glucose management; otherwise, persistent infection will continuously reactivate the hyperglycemia-driven pathological cascade, undermining the benefits of metabolic intervention.

### Promotion of angiogenesis

Angiogenesis, the formation of new blood vessels from preexisting vasculature, is essential for DWs healing, as it restores tissue perfusion and ensures the delivery of oxygen and nutrients to the ischemic wound bed [142]. However, in the diabetic milieu, this process is profoundly impaired by chronic oxidative stress, persistent bacterial infection, and dysregulated cellular signaling [143–145]. Nanomaterial-based strategies have emerged as a powerful approach to overcome these barriers, primarily by enabling the targeted delivery and controlled regulation of key angiogenic mediators, as detailed in this section.

A central mechanism by which nanomaterials foster angiogenesis in DWs is through the enhanced expression, secretion, or spatially localized delivery of pivotal angiogenic factors, most notably VEGF and fibroblast growth factor [146–148]. These soluble mediators act as potent signaling cues that drive the proliferation, migration, and capillary-like tube formation of VECs, thereby re-establishing functional microcirculation within the wound.

Before examining specific nanotherapeutic strategies, it is important to acknowledge a methodological limitation that pervades the current preclinical literature. The vast majority of studies evaluating angiogenesis in DWs rely on surrogate endpoints: most commonly, VEGF expression levels, CD31 immunohistochemical staining, or in vitro tube-formation assays performed on Matrigel. While these indices provide useful evidence of endothelial cell activation and proliferative capacity, they do not, in themselves, constitute proof of functional, mature, and perfused vasculature. CD31 labels endothelial cells irrespective of vessel maturity, and an increase in vessel density quantified by CD31^+^ area may reflect a disorganized plexus of immature, leaky, and nonperfused capillaries rather than a genuinely restored microcirculation [149]. Similarly, tube-formation assays measure the intrinsic ability of endothelial cells to form cord-like structures under highly artificial conditions; they fail to recapitulate the complex 3-dimensional architecture, hemodynamic forces, and pericyte-supported environment that define functional vessels in vivo. Therefore, although the following sections summarize studies that have employed these widely used metrics, their findings should be interpreted with caution: enhanced CD31 expression or VEGF up-regulation is indicative of pro-angiogenic signaling but must be distinguished from the re-establishment of stable, hierarchically organized, and functional vascular networks that ultimately support tissue perfusion and wound closure. Whenever possible, we highlight studies that incorporate more rigorous orthogonal assessments, such as direct perfusion assays (e.g., lectin perfusion or Doppler imaging), pericyte coverage analysis (e.g., NG2 or α-SMA costaining), or quantitative vascular permeability measurements, as these approaches provide considerably stronger evidence for meaningful functional vascular regeneration.

**Fig. 8.**
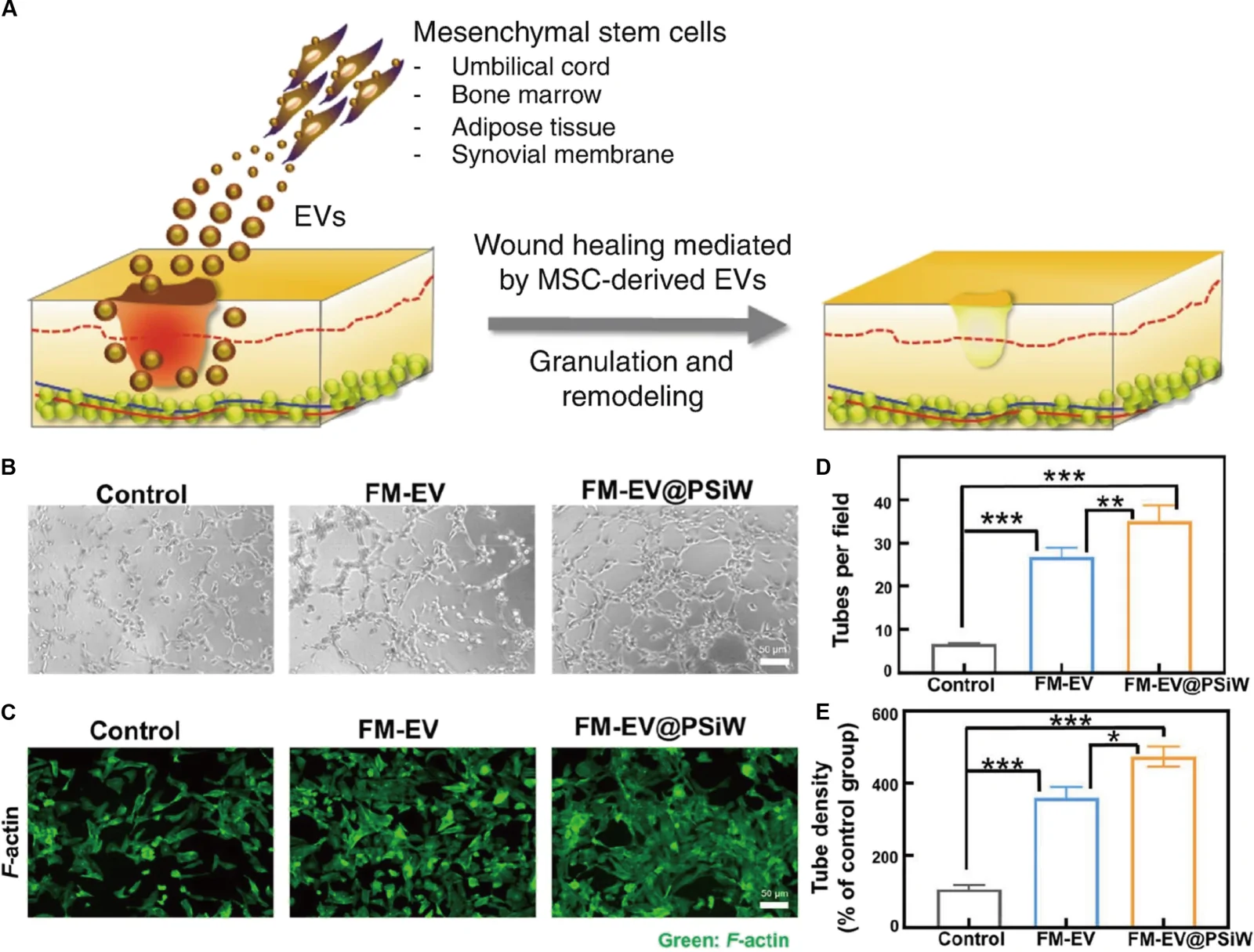
The trophic role of MSC-EVs in wound healing. (A) Studies have shown that EVs secreted by MSCs are capable of enhancing wound repair in various preclinical models. Reproduced with permission [150]. Copyright 2021, Springer Nature. (B to E) Angiogenesis capacity of FM-EV@PSiW. Reproduced with permission [157]. Copyright 2024, Elsevier.

Among the most promising nanoscale platforms for the delivery of angiogenic factors are EVs derived from mesenchymal stem cells (MSCs) (Fig. 8A) [150]. MSC-EVs inherit the intrinsic regenerative properties of their parent cells, encapsulating a multifaceted cargo of microRNAs, proteins, and lipids that collectively stimulate the migration of VECs, suppress apoptosis of key wound-resident cells, and resolve chronic inflammation, thereby synergistically accelerating tissue repair [150–152]. Preclinical studies consistently demonstrate that MSC-EVs effectively counteract multiple impediments to angiogenesis in DWs, including oxidative stress-induced VEC dysfunction [153–155]. Moreover, NO has been shown to enhance the secretion of EVs from MSCs, accompanied by an increase in VEGF expression [156]. Capitalizing on this mechanism, environment-responsive nanomaterials have been engineered to release NO through chemical reactions, thereby promoting angiogenesis in a controlled manner.

The therapeutic efficacy of MSC-EVs is critically influenced by the tissue origin of the parental MSCs. Although MSCs can be isolated from diverse sources, including adipose tissue, scalp, and facial skin, the angiogenic potency of their derived EVs varies considerably depending on the tissue of origin. To exploit a particularly potent source, Xu et al. [157] develop an injectable hydrogel loaded with EVs derived from human foreskin MSCs (FM-EVs), a relatively underexplored but highly angiogenic lineage. Their study demonstrates that FM-EVs substantially outperform EVs from conventional sources in promoting endothelial tube formation (Fig. 8B to E). To further augment their therapeutic impact, the authors engineer the FM-EVs with a polyvinylpyrrolidone (PVP) coating, a biocompatible polymer that enhances EV stability, prolongs retention at the wound site, and amplifies paracrine signaling, including the sustained release of VEGF. By integrating PVP-functionalized FM-EVs within a hydrogel matrix, this platform enables the localized and durable delivery of highly bioactive EVs while simultaneously addressing the critical translational challenge of EV instability, thus representing a profound advance toward clinical application.

Despite their therapeutic promise, the clinical translation of MSC-EVs for DWs faces formidable challenges spanning production, quality control, and regulatory pathways. Biological source variability, including donor health status, tissue origin, and culture conditions, generates substantial cargo heterogeneity that undermines batch-to-batch consistency and therapeutic potency [158,159]. Scalable isolation methods such as ultracentrifugation, tangential flow filtration, and size-exclusion chromatography each present trade-offs among purity, yield, and Good Manufacturing Practice (GMP) compatibility, while the absence of standardized potency assays capable of reliably predicting in vivo efficacy further complicates product characterization [160,161]. Physicochemical instability during storage, together with unresolved issues concerning optimal dosing regimens, administration frequency, and potential immunogenicity, adds critical translational uncertainties [162]. Finally, the ambiguous regulatory classification of EVs and the lack of specific guidelines create undefined approval pathways, collectively impeding the progression of EV-based therapies from bench to bedside.

Researchers have addressed these bottlenecks by developing an engineered biomimetic nanovesicle (NV) platform based on physical disruption and membrane extrusion. By subjecting therapeutic cells, including MSCs, macrophages, or endothelial cells, to ultrasonic disruption, high-pressure homogenization, or serial extrusion through porous membranes, cell membrane-derived vesicles with controlled diameters (typically 100 to 300 nm) are directly obtained [163]. Compared with natural EVs, these engineered NVs offer several distinct advantages.

First, in terms of yield and scalability, engineered NVs are produced at yields several orders of magnitude higher than those of natural EVs. For example, Yao et al. [164] prepare adipose-derived stem cell NVs (ADSC-NVs) with a yield exceeding 30-fold that of natural ADSC-EVs. Critically, the extrusion or ultrasonication-based preparation process does not depend on the time-consuming cellular secretion phase and is therefore far more amenable to industrial-scale manufacturing. Second, the membrane protein composition can be manually controlled. Unlike natural EVs, whose membrane protein profiles are dictated by complex intracellular sorting mechanisms, the protein repertoire of engineered NVs directly reflects the total membrane constituents of the parent cell. More importantly, cell membranes and liposomes from different sources can be mixed at defined ratios prior to extrusion, enabling the construction of heterologous membrane vesicles with dual functionalities. For instance, comixing M2 macrophage membranes with DOPE-pp-HBSP yields vesicles that simultaneously possess immunoregulatory and pro-angiogenic activities [165]. Third, exogenous drug loading can be precisely regulated. During the cell disruption and vesicle self-assembly process, high-concentration therapeutic molecules, such as VEGF, NO donors, pro-angiogenic small molecules, or GOx, can be directly encapsulated within the vesicle lumen. Zhang et al. [166] prepare endothelial cell NVs from induced pluripotent stem cells using an extrusion method, achieving efficient drug encapsulation and endothelial-targeted delivery. In contrast to the inefficient loading strategies that rely on transfection or coincubation of natural EVs, engineered NVs permit quantitative drug loading, and the loading amount can be precisely tuned by adjusting the feeding ratio. Fourth, batch-to-batch functional reproducibility is substantially improved. The heterogeneity of natural EVs arises from dynamic fluctuations inherent in the cellular secretion process, whereas the starting material for engineered NVs (cell lysates) and the physical fabrication process can be rigorously standardized [163]. By implementing a 3-tier quality control system: (a) particle size and charge consistency assessed by nanoparticle tracking analysis, (b) quantitative proteomic monitoring of key membrane markers such as CD9, CD63, CD81, and functional proteins, and (c) functional batch-release testing of in vitro angiogenic activity, batch-to-batch variability can be markedly reduced, thereby meeting the fundamental requirements of regulatory agencies for product homogeneity. Collectively, engineered biomimetic NVs, produced through a top-down physical disruption strategy, fundamentally circumvent limitations of natural EVs such as the heterogeneity. They offer a more controllable, scalable, and GMP-compatible path toward the clinical translation of pro-angiogenic therapies for DWs.

**Fig. 9.**
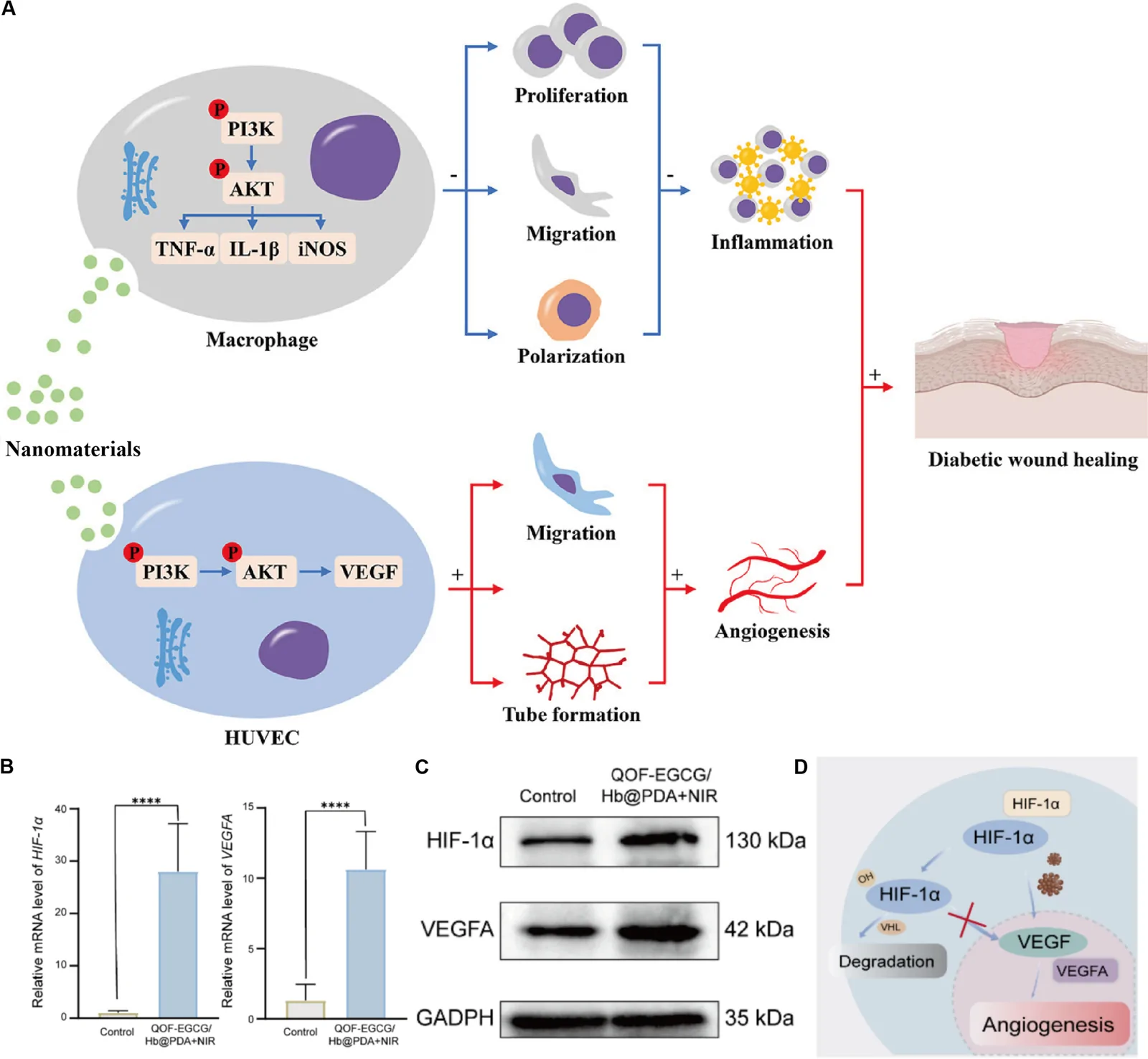
Nanomaterials facilitate angiogenesis through certain signaling pathways. (A) Nanomaterials promote phosphorylation of PI3K, then phosphorylation of AKT, thereby up-regulate the level of VEGF and promote angiogenesis. Adapted with permission [170]. Copyright 2025, Elsevier. (B to D) Under a hypoxic environment, HIF-1α bypasses down-regulation, directly enters the nucleus, and promotes the transcription of VEGF, thereby facilitating angiogenesis. Reproduced with permission [176]. Copyright 2025, Elsevier.

Angiogenesis is orchestrated by evolutionarily conserved intracellular signaling cascades, with the PI3K–Akt (Fig. 9A) and HIF-1α–VEGF (Fig. 9B to D) axes serving as central regulators of VEC proliferation, migration, and vascular morphogenesis [167,168]. In DWs, however, chronic hyperglycemia, oxidative stress, and impaired oxygenation synergistically perturb these signaling networks, culminating in profound deficits in vascular regeneration [169]. In response, nanomaterial-based strategies have been rationally engineered to selectively reactivate these compromised pathways and restore their pro-angiogenic output.

The PI3K–Akt signaling pathway is a pivotal node in angiogenic regulation, governing essential cellular functions such as survival, metabolism, and cytoskeletal remodeling [170,171]. In the context of diabetes, this pathway is frequently attenuated, thereby impairing VEC activation and neovascularization [172]. Zhou et al. [173] provided direct experimental validation of this concept using EVs derived from adipose-derived stem cells (ADSC-EVs), natural nanocarriers endowed with intrinsic regenerative properties. Their work demonstrated that ADSC-EVs administration markedly up-regulates CCN2 expression at the wound site. Crucially, functional assays revealed a causal link: both genetic knockdown of CCN2 and pharmacological inhibition of the PI3K–Akt pathway substantially abrogated the pro-angiogenic effects of ADSC-EVs. These findings collectively establish that ADSC-EVs drive DWs healing chiefly through activation of the CCN2–PI3K–Akt signaling axis, thereby bridging stem cell-derived nanotherapeutics with precise molecular reactivation of angiogenesis.

HIF-1α is a master transcriptional regulator of cellular adaptation to hypoxia and plays a nonredundant role in driving angiogenesis [174,175]. Under normoxic conditions, HIF-1α is constitutively hydroxylated by prolyl hydroxylases (PHDs), a modification that targets it for ubiquitination and rapid proteasomal degradation. In DWs, however, persistent hypoxia inhibits PHD activity, leading to HIF-1α stabilization and cytoplasmic accumulation. The stabilized HIF-1α translocates to the nucleus and recruits transcriptional coactivators to form a complex that binds hypoxia response elements (HREs) in the promoters of target genes. This transcriptional program is dominated by the induction of key angiogenic mediators, most notably VEGF [176]. Therefore, promoting HIF-1α stabilization has emerged as a core therapeutic strategy to drive angiogenesis in DWs. Ge et al. [177] leveraged this principle through a nanomaterial-based approach employing tetrahedral framework nucleic acids (tFNAs), self-assembled DNA nanostructures renowned for their biocompatibility, structural precision, and high cellular uptake. They demonstrated that tFNA treatment substantially up-regulates HIF-1α levels in DWs. Mechanistically, the accumulated HIF-1α binds to HREs within the VEGF promoter, driving its transcription and secretion. The elevated VEGF levels then stimulate VEC proliferation, migration, and tube formation, thereby accelerating vascular regeneration and wound healing.

However, a critical distinction must be drawn between VEGF-driven endothelial proliferation and the establishment of functional, mature vasculature. VEGF is a potent initiator of angiogenesis; however, its sustained or excessive expression, particularly in the setting of HIF-1α dysregulation, can generate abnormal, tortuous, and hyperpermeable vessels that are poorly perfused and fail to deliver adequate oxygen and nutrients [178]. Such immature vessels are characterized by insufficient pericyte coverage, which leads to structural fragility and leakage, paradoxically exacerbating edema and inflammation within the wound bed rather than facilitating healing. Furthermore, VEGF-induced angiogenesis, in the absence of concomitant ECM remodeling and appropriate hemodynamic integration, frequently results in a chaotic, nonhierarchical vascular network that does not restore functional microcirculation. Thus, although up-regulation of HIF-1α and VEGF represents a necessary initial step in stimulating neovascularization, it is insufficient to conclude that functional microcirculation has been re-established. A more rigorous evaluation framework is required, one that incorporates the assessment of vessel perfusion (e.g., via intravenous lectin or fluorescent microsphere injection), vessel maturity (pericyte coverage index using NG2/α-SMA and CD31 costaining), vascular permeability (Evans blue extravasation or FITC-dextran leakage), and the hierarchical organization of the vascular network (3-dimensional imaging of vessel branching patterns). To date, very few nanomaterial studies have integrated these functional endpoints, representing a critical gap in the current evidence base.

Moreover, it is important to recognize that, although these pathways are predominantly beneficial in the context of DWs, their prolonged or uncontrolled activation carries the risk of generating abnormal, immature, or hyperpermeable vessels that fail to restore functional perfusion and may paradoxically impair wound resolution [179]. For instance, sustained, excessive VEGF expression driven by unabated HIF-1α stabilization can produce chaotic, leaky neovasculature that exacerbates inflammation and edema [180]. Both the PI3K–Akt and HIF-1α–VEGF axes operate within finely tuned signaling networks, and their ultimate effects are highly context-dependent, being shaped by factors such as the specific cell types involved, the dosage and pharmacokinetics of the nanotherapeutic, the timing of intervention, and the stage of disease progression. To overcome the barrier, researchers have developed strategies to achieve spatiotemporally controlled and self-limiting activation of these pathways, recapitulating the dynamics of physiological angiogenesis rather than providing a persistent pro-angiogenic stimulus. A representative example is the injectable nanocomposite hyaluronic acid hydrogel system (HA-DDP/NPs), which enables temporally controlled release of VEGF for early endothelial sprouting and platelet-derived growth factor-BB (PDGF-BB) for late-stage pericyte recruitment and vessel stabilization [181]. This design recapitulates the natural spatiotemporal cascade of vascular maturation, avoiding the pitfalls of simultaneous factor administration. In DW models, such sequential platforms have been shown to promote perfused, mature vasculature with improved functional integration, rather than leaky and immature capillary networks.

A critical yet frequently overlooked translational consideration in the design of pro-angiogenic nanomaterials is the timing of therapeutic intervention. VEGF is a potent initiator of endothelial proliferation; however, when its activation is sustained or occurs prematurely while the wound bed remains actively inflamed and burdened by elevated oxidative stress, it paradoxically drives the formation of aberrant, tortuous, and hyperpermeable neovessels. These immature vessels typically lack adequate pericyte coverage, fail to restore functional microcirculation, and can exacerbate edema and tissue exudation rather than improve nutrient and oxygen delivery. Therefore, the therapeutic window for angiogenic nanomaterials must be carefully aligned with the resolution of upstream pathological drivers. Drawing on the 3-phase model, we suggest that robust pro-angiogenic stimulation, for example, via VEGF-overexpressing EVs, HIF-1α-stabilizing DNA frameworks, or growth-factor-loaded hydrogels, should be preferentially initiated during the late phase (days 7 to 14). At this stage, local glucose levels have been normalized, biofilms eradicated, and the oxidative and inflammatory burden substantially reduced. In contrast, early-phase VEGF up-regulation (days 0 to 3) risks generating a chaotic vascular plexus that lacks hierarchical organization and functional perfusion, ultimately delaying rather than accelerating wound closure.

To implement such temporal precision, future nanoplatforms should incorporate feedback-responsive release mechanisms. For instance, angiogenic cargoes could be engineered to be unlocked only when local ROS concentrations fall below a predefined threshold, or when MMP activity signals the transition to the remodeling phase. This approach ensures that vascular regeneration is initiated precisely when the microenvironment becomes permissive for stable, mature vessel formation. Such “on-demand” timing logic is essential to distinguish genuine restoration of perfusion from mere endothelial hyperplasia, and represents a critical step toward translating pro-angiogenic nanotherapies into clinically meaningful wound care.

### Immune regulation

In DWs, hyperglycemia-driven oxidative stress sustains a chronic, nonresolving inflammatory state that arrests the healing cascade. The immune landscape of these wounds is characterized not merely by a skewed macrophage polarization, but by a broader dysfunction spanning both innate and adaptive immunity, including aberrant neutrophil infiltration, dysregulated T cell responses, and impaired efferocytosis of apoptotic cells. Critically, the wound healing process demands a precisely timed immune response: robust innate activation is essential for early pathogen control, yet its timely resolution is equally critical for the transition to tissue repair. Overly aggressive or premature suppression of inflammation can compromise antimicrobial defense and prolong infection. To navigate this therapeutic balance, nanomaterial-based strategies are being rationally designed to achieve temporally controlled, context-responsive immunomodulation—restoring immune homeostasis through mechanisms that extend beyond macrophage repolarization to encompass the modulation of neutrophil activity, the active promotion of inflammatory resolution, and the orchestration of adaptive immune components.

**Fig. 10.**
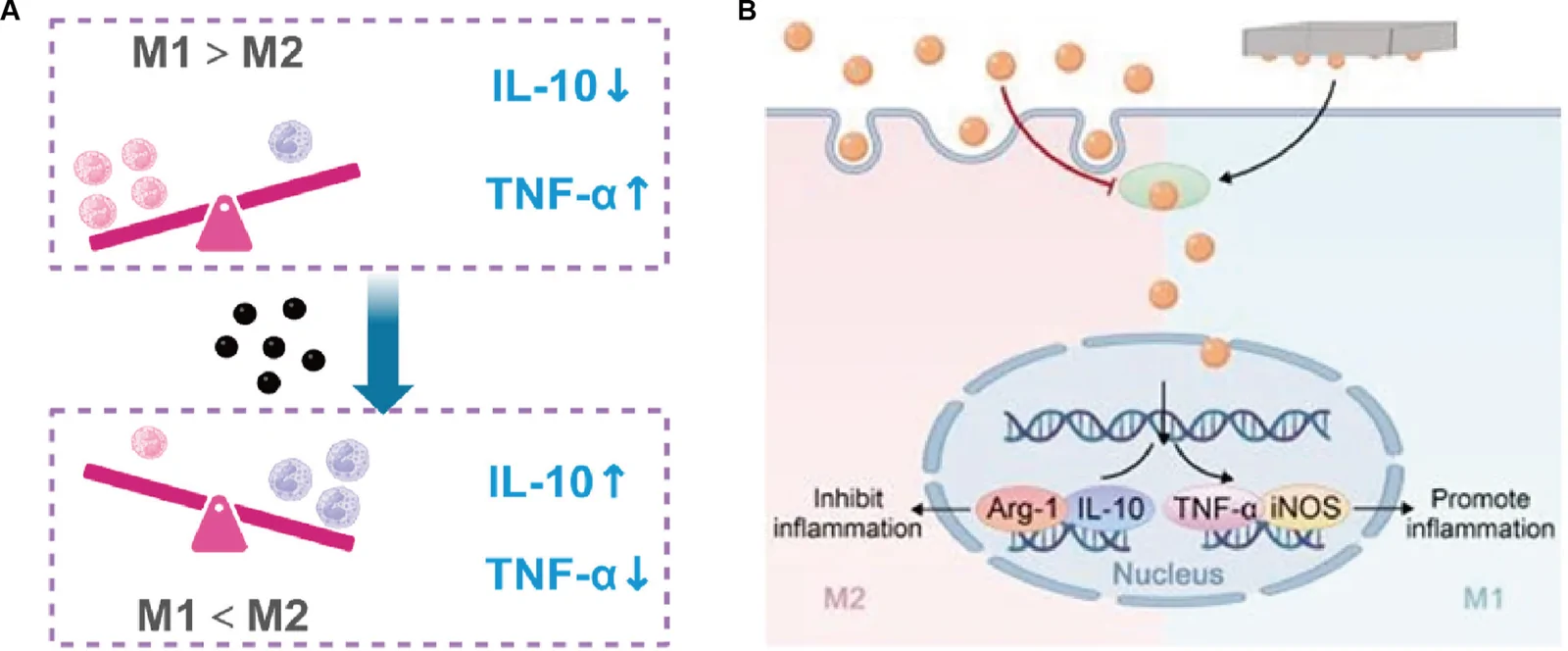
Consequences of immune regulation of nanomaterials toward the microenvironment of DWs by promoting macrophage polarization. (A) Nanomaterials have the capacity to facilitate transfer of macrophages to M2 type. Adapted with permission [222]. Copyright 2025, KeAi. (B) Expression of inflammatory factors is inhibited by nanomaterials. Reproduced with permission [263]. Copyright 2025, Springer Nature.

Macrophages serve as central orchestrators of the wound immune landscape, capable of adopting functionally opposing phenotypes that shape the inflammatory trajectory. The classically activated M1 phenotype initiates acute inflammation through pro-inflammatory cytokines (e.g., TNF-α and iNOS) (Fig. 10); however, its prolonged persistence in DWs causes collateral tissue damage and impedes repair [182]. In contrast, the alternatively activated M2 phenotype dampens inflammation and promotes regeneration by releasing anti-inflammatory cytokines (e.g., IL-10) and growth factors that stimulate angiogenesis, fibroblast proliferation, and ECM remodeling [183]. In DWs, this equilibrium is skewed toward sustained M1 dominance, creating a self-perpetuating cycle of inflammation that actively suppresses healing [184]. Consequently, a central objective in nanomedicine has been to develop platforms that reprogram macrophage polarization from M1 to M2, thereby reinstating a pro-regenerative milieu [185]. However, M2 polarization is not universally beneficial [186]. It has been proved that c-Maf-positive M2 macrophages can drive fibroblast activation and excessive ECM deposition, thereby promoting hypertrophic scar formation and fibrosis rather than regeneration [187]. Therefore, therapeutic strategies toward DWs should aim not at simply driving an M1-to-M2 transition but at restoring the spatiotemporal coordination of macrophage functions, effectively resolving inflammation while preserving host defense and avoiding fibrotic sequelae.

Moreover, it is important to recognize that the classical M1/M2 dichotomy, although conceptually useful, substantially oversimplifies the true complexity of macrophage biology in wound healing. In vivo, macrophages do not exist as discrete M1 or M2 endpoints but instead exhibit a continuous spectrum of activation states that are dynamically shaped by the evolving wound microenvironment [188]. Single-cell transcriptomic and epigenomic studies have revealed that wound macrophages frequently coexpress markers traditionally assigned to both M1 and M2 categories and can shift between functional programs without passing through a binary switch [189]. Beyond heterogeneity, macrophages exhibit distinct spatiotemporal dynamics that are essential for normal wound repair. During the early phase of healing, pro-inflammatory macrophages predominate and are responsible for eliminating pathogens and clearing necrotic debris; subsequently, macrophages transition to a pro-reparative phenotype that secretes growth factors to promote angiogenesis and tissue remodeling [190]. This temporally coordinated phenotypic switch is critical for successful healing. In DWs, however, this spatiotemporal program is severely disrupted: macrophage infiltration itself is substantially delayed, and once recruited, pro-inflammatory macrophages persistently accumulate and fail to execute the transition to a pro-reparative state even at late time points [190]. Epigenetic regulation plays a central role in this pathological arrest. Studies demonstrate that the histone demethylase JMJD3 is markedly up-regulated in macrophages within DWs, driven by the JAK1/3–STAT3 signaling axis, and that JMJD3 sustains the ongoing transcription of inflammatory genes [191]. JMJD3 also regulates the expression of STING, which, in the diabetic context, acts to restrain wound closure. Consequently, the aberrant epigenetic reprogramming of macrophages represents both a mechanistic hallmark of impaired healing and a potential therapeutic target.

To address the heterogeneity of macrophages and the disruption of their spatiotemporal dynamics, nanomaterials offer a precise interventional toolkit. As for the heterogeneity of macrophages, nanomaterial-based strategies are now being rationally designed not to simply “witch” this binary state, but to deliver precise, engineered molecular signals that navigate macrophages along this continuum toward a pro-regenerative endpoint. For instance, IL-4 and IL-10 are well-established inducers of the M2-like phenotype, acting through the JAK-STAT6 and STAT3 pathways [192,193], respectively. However, their clinical utility is limited by short half-lives and systemic adverse effects. To address this limitation, these cytokines have been encapsulated within nanocarriers, such as poly (lactic-co-glycolic acid) (PLGA) nanoparticles or mesoporous silica nanoparticles, which protect the payload from rapid degradation and enable spatiotemporally controlled release directly within the wound bed [194,195]. This sustained, localized cytokine signal provides a continuous, instructive cue to infiltrating macrophages, effectively overriding the inflammatory milieu and driving their phenotypic transition toward a reparative state characterized by elevated arginase activity and the secretion of tissue-remodeling factors. Beyond these cytokines, another prominent example is the surface decoration with phosphatidylserine (PS). In the context of apoptotic cell clearance, surface-exposed PS acts as a classic “eat-me” signal, engaging the TAM receptor tyrosine kinase family (Tyro3, Axl, and Mer) on macrophages [196]. This engagement triggers a signaling cascade that not only promotes efferocytosis but also actively suppresses NF-κB-dependent inflammatory transcription while up-regulating the expression of anti-inflammatory mediators like transforming growth factor-β (TGF-β) and IL-10. Translating this to nanomedicine, several studies have now engineered PS-containing liposomes or PS-coated polymer nanoparticles. For instance, Zhang et al. [197] developed soft, deformable PS-containing nanoliposomes (d-PSLs) that persistently bind to macrophage membranes and promote M2-like polarization, substantially accelerating wound closure in diabetic rats. Similarly, Kraynak et al. [198] constructed apoptotic body-inspired nanoparticles (PS-MNPs) by coating PLGA cores with PS-supplemented cell membranes, which reduced TNF-α secretion by 5-fold and suppressed NF-κB activation in LPS-challenged macrophages. Notably, lipid-polymer nanoparticles containing PS are preferentially taken up by M2-polarized macrophages (up to 166-fold higher than PS-free counterparts), whereas PS-free nanoparticles are preferentially internalized by M1-polarized macrophages. Upon administration to DWs, these PS-mimetic nanoparticles preferentially bind to Mer receptors on infiltrating macrophages. This ligand–receptor interaction acts as a powerful, noncytokine “instruction manual” that drives macrophages to adopt an M2-like state without the need for internalization or the release of any toxic agent.

As for the disruption of the spatiotemporal dynamics of macrophages, researchers have designed macrophage-specific nanoparticles that inhibit JMJD3, substantially reducing the levels of inflammatory cytokines and STING and effectively improving the repair of DWs building on the aberrant up-regulation of JMJD3 in DW macrophages identified through single-cell RNA sequencing (scRNA-seq) [191]. This strategy exemplifies a research paradigm in which pathological mechanisms uncovered at single-cell resolution are directly translated into targeted nanomaterial interventions. Additionally, biomaterials with temporally programmed functionality are gaining attention: for example, the temporally programmed release of pro-resolving mediators restores the impaired efferocytic capacity of macrophages in DWs, thereby re-establishing the temporal rhythm of inflammation resolution [199]. Collectively, these studies indicate that the design of future nanomaterials should not merely pursue the singular goal of promoting M2 polarization. Rather, it should aim to act at the appropriate time, with the appropriate intensity, on the appropriate macrophage subpopulations, in order to reconstruct the spatiotemporal order of wound healing.

While the studies discussed above predominantly rely on conventional methods like qPCR and flow cytometry to assess a select panel of M1/M2 markers, the field is increasingly recognizing the need for more comprehensive validation to truly capture the complexity of macrophage reprogramming. Recent advances in high-dimensional single-cell technologies, such as scRNA-seq and mass cytometry (cytometry + time of flight, CyTOF), now enable an unbiased, global profiling of macrophage heterogeneity at the single-cell level [200,201]. Furthermore, proteomic and phosphoproteomic analyses can reveal posttranscriptional and posttranslational regulatory events that are critical for functional phenotypic shifts [202]. Integrating these multi-omics approaches is essential to move beyond phenomenological descriptions of M1/M2 marker changes and toward a mechanistic understanding of how nano-bio interactions shape the entire macrophage functional continuum. This paradigm shift will be critical for the rational design of next-generation immunomodulatory nanomedicines.

The immune response in DWs is profoundly and persistently dysregulated, extending well beyond the widely recognized M1/M2 macrophage imbalance. Neutrophils are the first innate immune effector cells recruited to the site of injury during wound healing [203]. Under homeostatic conditions, they defend against invading pathogens through phagocytosis, degranulation, and the formation of NETs. In DWs, however, neutrophil function is profoundly compromised. The hyperglycemic milieu markedly impairs neutrophil chemotaxis and oxidative burst capacity, resulting in delayed recruitment to the wound bed. More critically, neutrophils in DWs exhibit a dysregulated NETosis phenotype: the pentose phosphate pathway, which is essential for neutrophil effector functions, is up-regulated, leading to increased production of NADPH, which, in turn, drives the generation of large amounts of ROS, a key signaling molecule that induces NETosis [204]. Specifically, NETosis causes robust accumulation of NETs, which inhibit fibroblast viability and collagen synthesis; they induce endothelial-to-mesenchymal transition via the Hippo signaling pathway, thereby impairing angiogenesis; they suppress macrophage efferocytosis through the PI3K/Rac1 signaling axis, preventing the timely clearance of apoptotic cells; and they perpetuate a chronic inflammatory state that further delays tissue repair [205]. Additionally, NETs can exacerbate fibroblast dysfunction by inducing ferroptosis [206]. Collectively, these derangements establish a self-perpetuating cycle of inflammation and impaired healing that must be targeted by effective therapeutic strategies.

Nanomaterial-based strategies targeting neutrophil dysfunction are emerging as an active area of investigation. Broadly, these strategies can be categorized into 2 complementary approaches: those that directly modulate neutrophil function and those that eliminate or degrade preformed NETs. In the direct-targeting category, potassium-doped manganese oxide nanoparticles (K-MnO_2_ NPs) induce neutrophil depolarization through electron transfer, which activates transmembrane Ca^2+^ channels and triggers Ca^2+^ influx, thereby reprogramming the Ca^2+^-dependent behavior of dysfunctional neutrophils [207]. This strategy restores chemotaxis, ROS production, and apoptotic clearance, and has demonstrated potent antibacterial and pro-healing effects in a DW model infected with MRSA. In a complementary approach, supramolecular self-assembled IGF-1 nanofibers (Nap-FFG-IGF-1) suppress excessive NET formation by inhibiting the NF-κB signaling pathway, while concurrently promoting tissue regeneration [208]. With respect to NET clearance, deoxyribonuclease I (DNase I) degrades the DNA backbone of NETs [34]. However, its clinical utility is limited by a short circulating half-life and potential cytotoxicity. To overcome this, self-assembled nanocomplexes of DNase I coassembled with low-molecular-weight polyphenols (O/DNase-I) have been developed, achieving efficient NET clearance and accelerated wound healing in DWs [209]. MSC-derived exosomes, particularly those engineered to carry miR-92a-1-5p, inhibit NET formation through the S100A9 pathway, and exosomes derived from hypoxia-preconditioned MSCs similarly alleviate excessive NETosis [210]. Glucose-responsive hydrogels (CF-CPGaMPN) have also been shown to suppress NET formation and ameliorate the hypoxic cellular microenvironment [211]. Beyond the 2 approaches, a more innovative strategy exploits the divergent fates of neutrophils for precision intervention. Inspired by the concurrent accumulation of apoptotic neutrophils and excessive NETs in DWs, a composite platform comprising exosomes and a hydrogel loaded with IL-4 mRNA has been developed [212]. This system harnesses the preexisting pool of apoptotic neutrophils to amplify pro-healing gene expression and rescue the necroptosis defect induced by excessive NETosis, thereby achieving branch-specific modulation of neutrophil fate. This represents a paradigm shift from indiscriminate inhibition toward fate-based precision regulation.

Collectively, neutrophils play a complex dual role in DWs: they constitute the first line of antimicrobial defense, yet are also key drivers of chronic inflammation and tissue injury. Future nanomaterial design should therefore transcend the simple inhibition of NETosis and instead be grounded in a detailed understanding of the divergent branches of neutrophil fate, aiming to precisely modulate neutrophil recruitment, activation, and clearance within the appropriate temporal windows. Only through such spatiotemporally precise regulation can the antibacterial functions of neutrophils be preserved while avoiding tissue damage arising from their excessive activation.

Dendritic cells (DCs) serve as immune sentinels in the skin. During normal cases, they activate naïve T cells through antigen presentation, thereby initiating adaptive immune responses [213]. In the hyperglycemic microenvironment of diabetes, however, DC function is severely compromised. A prominent manifestation of this dysfunction is impaired efferocytosis—the phagocytic clearance of apoptotic cells, which is essential for maintaining tissue homeostasis, suppressing inflammation, and promoting repair [32]. In DWs, aberrantly elevated expression of the SLC7A11 membrane transporter inhibits the efferocytic activity of DCs, leading to the persistent accumulation of apoptotic cells [214]. These uncleared cells release autoantigens and damage-associated molecular patterns (DAMPs), further amplifying inflammatory signals and perpetuating a vicious cycle [32]. Single-cell transcriptomic analyses further demonstrate that the number of monocyte-derived DCs is markedly reduced in diabetic mouse wounds, a reduction that correlates closely with delayed wound closure [215]. Mechanistically, the transcription factor IRF4 plays a critical role in DC differentiation: loss of IRF4 impairs monocyte-to-DC differentiation, resulting in the sustained accumulation of pro-inflammatory monocytes and macrophages at the wound site. Importantly, the reduction in DC numbers also diminishes the population of activated regulatory T cells (Tregs), suggesting that DCs may indirectly facilitate wound healing through the modulation of Tregs [215].

Nanomaterial-based strategies targeting DC dysfunction are now emerging. For instance, nanofiber dressings loaded with nanozymes are designed to enhance DC efferocytosis by inhibiting SLC7A11 [214]. In a complementary approach, nanoparticles coated with apoptotic neutrophil membranes are developed to augment DC efferocytic function through a dual-pathway intervention [216]. Exosomes derived from TNF-α-preconditioned adipose-derived MSCs (T-exos) inhibit excessive DC activation through the miR-146a-5p/TXNIP/NLRP3 axis, substantially accelerating wound closure in diabetic mice in vivo [217]. Collectively, these studies establish that restoring normal DC function, particularly their efferocytic capacity, represents a critical strategy for re-establishing immune homeostasis in DWs.

T cells, as central effectors of adaptive immunity, exhibit profound subset imbalances in DWs. Among these, the imbalance between Tregs and Th17 cells within the CD4^+^ helper T cell compartment represents one of the most prominent pathological features. Tregs are key regulators that maintain immune homeostasis by expressing immunomodulatory molecules and secreting anti-inflammatory cytokines, thereby promoting inflammation resolution, angiogenesis, and tissue repair [218]. In skin wounds, skin-resident Tregs interact with both immune and nonimmune cells to contribute to the restoration of skin barrier integrity. In the diabetic state, however, both the number and suppressive function of Tregs are substantially reduced, a deficiency that closely correlates with delayed wound healing [219]. In contrast, Th17 cells drive inflammatory responses through the secretion of IL-17A [220]. In DWs, T cell differentiation is markedly skewed, with an elevated Th1/Th17 ratio and a diminished Treg compartment. This Treg/Th17 imbalance perpetuates chronic inflammation, increases susceptibility to infection, and impairs tissue repair [221]. Moreover, the reciprocal interaction between Th17 cells and macrophages may further entrench the vicious cycle of chronic inflammation and repair failure.

Based on an increasingly detailed mechanistic understanding of T cell dysregulation, nanomaterial-based strategies that specifically target T cell subsets are being actively developed. A representative example is provided by Zn-DHM nanozymes [222]. This metal-polyphenol nanozyme, synthesized through the coordination of Zn^2+^ with dihydrogen rosmarinic acid, combines efficient ROS scavenging and metabolic regulatory activities with a pronounced capacity to influence T cell differentiation. Studies demonstrate that Zn-DHM nanoparticles maintain the Th17/Treg balance by down-regulating the IL-17 signaling pathway to reduce inflammation, while simultaneously up-regulating FOXP3, the signature transcription factor of Tregs, to preserve immune homeostasis. Furthermore, Zn-DHM nanoparticles promote the differentiation of naïve CD4^+^ T cells into Tregs. The distinctive advantage of this strategy lies in its concurrent modulation of innate immunity (macrophage M1/M2 polarization) and adaptive immunity (Th17/Treg balance), thereby achieving comprehensive remodeling of the immune microenvironment. In parallel, advanced delivery systems have been designed to reinforce T cell subset regulation. A pH-responsive, dual dynamic cross-linked metal–polyphenolic hydrogel (CSp-OxD@Cu^2+^/G) forms a network through Schiff base and boronate ester bonds and gradually releases Cu^2+^/G nanoparticles in the acidic microenvironment of DWs, simultaneously reprogramming macrophages and restoring the Th17/Treg balance [221]. A biomimetic biotin-avidin hydrogel enables the sustained in vivo delivery of exosomes derived from stem cells from human exfoliated deciduous teeth (SHED-Exos), substantially lowering the Th17/Treg ratio and accelerating wound closure [223]. Additionally, a thermally responsive smart hydrogel (TGF-β1@MATH) integrates hollow mesoporous MnO_2_ nanozymes with TGF-β1 to scavenge ROS while promoting Treg recruitment and growth factor secretion [224].

The wound healing response is inherently temporally orchestrated: a robust initial pro-inflammatory phase is indispensable for pathogen clearance and the removal of necrotic debris [225]. Premature or excessive suppression of this phase can therefore impair bacterial eradication and delay subsequent healing. The resolution of inflammation is actively directed by specialized pro-resolving mediators (SPMs), including lipoxins, resolvins, and protectins, which counter-regulate the inflammatory cascade, promote the clearance of neutrophils, enhance macrophage efferocytosis, and facilitate a phenotypic transition toward reparative states, all without compromising host defense. Nanomaterials that deliver SPMs or functionally mimic their activity represent a promising yet underexplored strategy for achieving genuine inflammatory resolution in DWs. The research on using RvE1 encapsulated in synthetic targeted polymer nanoparticles for intestinal wound repair has demonstrated the feasibility of nanoparticle delivery of SPMs [226]. Beyond SPMs, restoring apoptotic function is the most direct intervention strategy, as we described previously. Nevertheless, the potential harm of prematurely suppressing antimicrobial inflammation must be explicitly acknowledged. Interventions that blunt early NF-κB or TNF-α signaling may increase susceptibility to wound infection, particularly given the high prevalence of MDR pathogens in diabetic ulcers. The robust neutrophil/M1 macrophage/Th1/Th17-driven response during the initial 0 to 3 d after wounding is indispensable for bacterial clearance, debris removal, and initiation of repair. Artificially enforcing M2 polarization or blunting pro-inflammatory signaling in this window, especially when biofilms persist or bacterial burden remains high, impairs phagocytic killing, delays efferocytosis of apoptotic neutrophils, and risks converting a healable ulcer into an intractable septic complication. Furthermore, excessive or untimely M2 skewing can also drive fibroblast hyperactivation and hypertrophic scarring.

Guided by the 3-phase model proposed previously, we therefore advocate that immunomodulatory nanomaterials are optimally deployed during the middle phase (days 3 to 7), after primary drivers such as local hyperglycemia, biofilm infection, and oxidative burden have been substantially mitigated. Crucially, this does not entail passive waiting; early-stage nanoplatforms can actively prime the immune system for subsequent resolution without prematurely suppressing host defense, for example, by delivering low-dose immunostimulatory signals that enhance pathogen killing without provoking a cytokine storm, or by incorporating scavengers that selectively remove DAMPs while preserving beneficial antimicrobial pathways. As the inflammatory milieu naturally subsides, the same platform or a sequentially applied second module can release pro-resolving mediators such as IL-10, SPMs, or TGF-β to orchestrate a controlled macrophage transition and promote tissue repair. Future nanomaterial designs should therefore incorporate microenvironment-sensing logic, such as bacterial toxin-activated antimicrobial modules paired with efferocytosis-triggered resolving modules, to ensure that immune reprogramming is initiated only after the infection risk has been neutralized. This approach reconciles the dual clinical imperatives of robust host defense and timely resolution while avoiding the fibrotic or immunosuppressive sequelae of indiscriminate or premature intervention.

### Intelligent closed-loop systems

The concept of “intelligent feedback integration” mentioned in the abstract has recently evolved from a theoretical design goal to tangible proof-of-concept systems capable of closed-loop behavior. These systems typically integrate 3 functional modules: (a) a sensing module that detects dynamic wound biomarkers; (b) a decision-making module that transduces biomarker signals into material state changes; and (c) an actuation module that executes on-demand therapeutic release.

For instance, Cheng et al. [227] developed a microenvironment-feedback hydrogel that leverages the alkaline pH of DW beds as “fuel” and GOx-catalyzed acid generation as “anti-fuel”, establishing a pH-glucose feedback loop that enables adaptive sol-gel cycling and programmable GOx release, achieving homeostatic wound pH and blood glucose levels in a type I diabetic mouse model. Similarly, MMP-9-responsive systems have been engineered to achieve enzyme-triggered on-demand drug release, taking advantage of the fact that MMP-9 is markedly overexpressed in chronic nonhealing wounds [228]. In a more integrated approach, Lin et al. [229] combined a NIR-responsive photothermal antibiotic-releasing hydrogel with a multichannel electrochemical biosensor array, creating a closed-loop patch capable of real-time monitoring of pH, uric acid, and glucose to guide on-demand drug administration. Notably, a battery-free, stretchable electronic wound bandage (iSAFE) has progressed to clinical trials in patients aged 18 to 95 with various wound types, demonstrating the translational potential of closed-loop systems [230].

Despite these advances, several challenges remain for practical implementation in the complex wound environment [231]. First, the wound milieu contains multiple interlinked biomarkers (pH, glucose, ROS, MMP-9, and bacterial load) that can interfere with each other, yet most current systems respond to only a single stimulus. Second, the long-term stability of integrated biosensors in the presence of wound exudate, enzymes, and bacteria remains a concern. Third, the transition from stimulus-responsive (“open-loop”) systems to truly closed-loop systems that feature continuous monitoring, real-time decision-making, and feedback-controlled intervention requires further integration of sensing and actuation functions. Finally, scalable manufacturing under GMP standards and regulatory pathways for such multifunctional devices remain undefined. Nonetheless, these proof-of-concept studies collectively demonstrate that intelligent feedback integration is no longer merely a theoretical goal but an actively evolving frontier in DW nanomedicine.

## Comparative Analysis with Other Advanced Wound Care Modalities

While the preceding sections have detailed the diverse nanotechnology-based strategies for DW microenvironment remodeling, a critical appraisal of how these emerging platforms compare with other advanced wound care modalities is essential to delineate their unique position in the therapeutic landscape. Recent literature has begun to address this comparative question directly, evaluating nanotechnology alongside bioengineered skin substitutes, growth factor therapies, and regenerative medicine modalities within unified analytical frameworks [232]. The emerging consensus is that no single therapeutic modality fully addresses the multifactorial pathological deficiencies of DW healing, and an evidence-based, patient-stratified, multimodal approach integrating bioengineering, molecular biology, nanotechnology, and regenerative medicine represents the optimal strategy for improving clinical outcomes.

Bioengineered skin substitutes represent one of the most established advanced wound care modalities. Leading products include Apligraf (a bilayered living cellular skin substitute containing both keratinocytes and fibroblasts), Dermagraft (a human fibroblast-derived dermal substitute), and EpiFix (a dehydrated human amnion/chorion membrane). These constructs function primarily by providing structural support and cellular components that promote re-epithelialization, granulation tissue formation, and ECM deposition [51]. The clinical evidence base for bioengineered skin substitutes is robust, supported by multiple randomized controlled trials (RCTs) and meta-analyses demonstrating superior wound closure rates compared to conventional dressings [233]. However, their mechanism of action is predominantly passive. They provide a structural template but lack the capacity to actively respond to dynamic changes in the wound microenvironment, such as fluctuating glucose levels, evolving infection status, or shifting oxidative stress burden. Their efficacy is compromised in infected wounds, as they lack intrinsic antibacterial properties. In direct comparison, nanotechnology platforms have demonstrated transformative antibacterial, antioxidant, and sustained drug delivery potential, which bioengineered skin substitutes do not possess. Nanomaterials can simultaneously address infection via nonantibiotic mechanisms (PTT, PDT, and metal ion release), regulate oxidative stress, and promote angiogenesis through modulation of signaling pathways such as HIF-1α–VEGF and PI3K–Akt, achieving multifactorial intervention within a single construct.

Recombinant growth factor therapies represent a molecularly targeted approach to DW management, with becaplermin (recombinant platelet-derived growth factor-BB, or rhPDGF-BB) being the sole FDA-approved molecularly targeted agent for DFUs [234]. PDGF-BB promotes wound healing through binding to PDGF receptors on target cells, stimulating chemotaxis and proliferation of fibroblasts, smooth muscle cells, and endothelial cells, as well as enhancing angiogenesis and ECM deposition. The clinical efficacy of becaplermin has been well documented: in a pivotal study, it achieved complete wound healing in 50% of patients versus 35% in the placebo group (*P* = 0.015) [235]. However, recombinant growth factor therapies face substantial practical challenges. The short half-life and inherent instability of growth factors in the proteolytic wound environment necessitate frequent application (typically once or twice daily), imposing heavy patient burden and cost. The therapeutic effect is limited to a single molecular target (the PDGF pathway), which cannot address the multifactorial nature of DW pathology encompassing infection, hyperglycemia, oxidative stress, and dysregulated inflammation. Furthermore, becaplermin’s efficacy is compromised in infected wounds, a frequent complication in DFUs. Nanotechnology offers a compelling alternative or complement to growth factor therapy. Nanoparticle-based delivery systems can encapsulate growth factors, enhancing their stability, prolonging their half-life, and enabling sustained, controlled release at the wound site. For example, lipid nanoparticles loaded with rhEGF have demonstrated higher fibroblast and keratinocyte proliferation and greater resolution of inflammation compared to free rhEGF [236]. Moreover, nanomaterials can independently trigger angiogenesis and collagen production through their intrinsic physicochemical properties, without requiring exogenous growth factors, a feature that distinguishes nanotechnology from growth factor therapies that depend entirely on exogenous protein administration.

Regenerative medicine modalities, including MSC therapy, exosome-based therapies, platelet-rich plasma (PRP), and gene therapy, have each demonstrated clinically meaningful benefits in refractory or high-risk DFUs [237]. MSCs promote wound healing through synergistic mechanisms including paracrine signaling, immunomodulation, neovascularization, and direct tissue regeneration. The therapeutic benefits of MSCs are increasingly attributed to their paracrine activity, particularly through EVs that carry bioactive microRNAs, proteins, and lipids. However, cell-based therapies face grave translational hurdles. Manufacturing complexity, high costs, batch-to-batch variability, stringent regulatory requirements, and challenges in maintaining cell viability and potency during storage and transport limit their widespread clinical deployment [238]. Additionally, the optimal cell source, dosage, and delivery route remain subjects of ongoing investigation [158,159]. Nanotechnology intersects with cell-based therapies in several important ways. First, nanoparticles coated with cell membranes, including MSC membranes, macrophage membranes, and exosome membranes, have demonstrated notable therapeutic effects in wound anti-inflammation and tissue regeneration. This biomimetic approach combines the targeting and immune-evasive properties of cell membranes with the drug-loading capacity and stability of synthetic nanoparticles. Second, stem cell-derived exosomes, which are themselves nanoscale vesicles, have emerged as highly promising cell-free therapeutic platforms because they preserve many regenerative and immunomodulatory properties of their parental stem cells while avoiding the safety concerns associated with live cell administration. Third, nanomaterials can serve as delivery vehicles for gene therapies, enabling targeted, sustained expression of therapeutic proteins such as growth factors directly within the wound bed [239].

The comparative literature reveals several key insights. First, bioengineered skin substitutes offer proven structural support with extensive clinical validation but lack active responsiveness and antibacterial capability. Second, growth factor therapies provide targeted molecular intervention but suffer from instability, single-target limitation, and compromised efficacy in infected wounds. Third, cell-based therapies harness the regenerative capacity of stem cells but face substantial manufacturing and regulatory hurdles. Nanotechnology platforms distinguish themselves through multifactorial targeting (antibacterial, antioxidant, pro-angiogenic, and immunomodulatory), intelligent responsiveness to wound microenvironment cues (pH, glucose, and ROS), sustained drug delivery that may reduce application frequency, and the capacity to serve as delivery platforms for growth factors, genes, and cellular products. However, a considerable translational gap between preclinical and clinical evidence persists. Unlike bioengineered skin substitutes and becaplermin, which have amassed extensive clinical trial data, most nanomaterial-based strategies remain at the preclinical or early clinical investigation stage. Critically, these modalities are not mutually exclusive. An evidence-based, patient-stratified, multimodal approach, including integrating bioengineering, molecular biology, nanotechnology and regenerative medicine, represents the optimal strategy for improving clinical outcomes in DFUs. Nanomaterials may serve not only as stand-alone therapeutics but also as delivery platforms to enhance the efficacy of growth factors, cellular products, and other bioactive agents, thereby realizing synergistic therapeutic benefits that transcend the capabilities of any single modality.

## Clinical Application and Future Challenges

Despite the proliferation of novel nanomaterial-based strategies in preclinical research, their translation into clinical practice has remained strikingly limited. To date, the only FDA-approved nanomaterial products for DW care are AgNP formulations, including Silvercel, SilverStat, and SilverSorb [98]. This stark disparity between experimental promise and clinical reality underscores the need to systematically examine the translational barriers, design trade-offs, and conceptual gaps that continue to impede the bench-to-bedside journey.

### Translational barriers

The transition from laboratory-scale nanomaterial development to clinical deployment is confronted by a series of interconnected hurdles that require systematic resolution.

1. Preclinical model limitations. The vast majority of efficacy and safety data for nanomaterials are derived from animal models that inadequately recapitulate the complexity of human DWs. Streptozotocin-induced diabetic rodents provide a rapid hyperglycemic background but predominantly model type 1 diabetes and heal largely by wound contraction, in stark contrast to the re-epithelialization-driven healing characteristic of human wounds [240]. Genetically diabetic models, such as db/db mice, better recapitulate sustained hyperglycemia, yet still rely heavily on contraction-mediated closure [241,242]. Infected and ischemic models introduce clinically relevant complications but oversimplify the polymicrobial nature and perfusion deficits observed in patients. Large animal models, including diabetic pigs, offer skin architecture and healing mechanisms more analogous to those in humans, but their high cost and limited availability restrict widespread use. Human skin equivalents and ex vivo tissues provide human-derived platforms, although they typically lack functional vasculature, systemic immune responses, and long-term metabolic dysregulation. Given these limitations, future research should prioritize advanced tissue-engineered constructs that integrate multiple human-relevant elements, such as immune components, perfusable vasculature, and sustained diabetic insults, to serve as a critical intermediate step between conventional animal studies and clinical trials.

2. Manufacturing scalability and quality control. Scaling nanomaterial platforms from benchtop to GMP production presents platform-specific hurdles that critically affect batch consistency, sterility, and stability. As for ligand-targeted liposomes, scale-up is hindered by poor size homogeneity and inconsistent ligand coupling efficiency. The polydispersity index often exceeds 0.2 upon a 5,000-fold increase. Standard methods such as microfluidics do not scale linearly, while 0.2-μm filtration and freeze-drying can cause particle loss, fusion, or drug leakage. These challenges necessitate extensive process controls and multivariate analysis, substantially raising production costs. As for metal-based nanoparticles, chemical reduction for AgNPs broadens the size distribution upon scale-up, altering both antibacterial activity and cytotoxicity. MOF assembly is exquisitely sensitive to mixing and temperature, leading to batch-to-batch variations in crystallinity, porosity, and drug loading. The high cost of organic linkers and solvents, combined with the need for green-synthesis innovations, remains unresolved for most novel MOF platforms. As for engineered EVs, upstream expansion in bioreactors introduces shear and oxygen gradients that shift the cellular secretome, yielding EV batches with variable miRNA and protein cargo [243]. Downstream, ultracentrifugation is not scalable, while tangential flow filtration, although scalable, typically recovers less than 30% of particles and may induce aggregation. The intrinsic heterogeneity of EVs further complicates the definition of critical quality attributes, and regulatory guidelines for acceptable variability are still lacking. As for enzyme-loaded nanoreactors (e.g., GOx@MOF), activity loss during scale-up results from enzyme aggregation and active-site masking caused by local concentration gradients. Cascade systems require re-optimization of enzyme ratios at each scale, and terminal sterilization (heat, ethylene oxide, or irradiation) is incompatible with enzyme stability, necessitating costly aseptic processing. Consequently, no such nanoreactor has yet entered clinical trials. Hydrogels suffer from nonuniform crosslinking during scale-up due to heat or light gradients, which alters drug release kinetics. MN molding faces incomplete cavity filling and drying-induced shrinkage, compromising needle integrity and skin penetration. Batch-to-batch variability in polymer molecular weight, together with poor storage stability (dehydration or moisture-softening), further complicates GMP manufacturing. Across all platforms, batch-to-batch consistency remains the foremost challenge, yet multistep nanofabrication makes reproducibility extraordinarily difficult. Sterility assurance is universally problematic because terminal sterilization often degrades nanomaterials, forcing reliance on expensive aseptic filling. Long-term stability and the absence of a unified regulatory framework for nanomedicines further extend development timelines. Addressing these hurdles will require continuous manufacturing, process analytical technologies, and close collaboration among materials scientists, engineers, and regulators.

3. Long-term biosafety and biocompatibility. Prolonged direct exposure of nanomaterials to damaged tissue raises a distinct set of safety concerns that differ fundamentally from those associated with conventional systemic drug administration. First, regarding nanoparticle accumulation in granulation tissue, their small size and high surface reactivity facilitate rapid uptake by macrophages and fibroblasts within the newly formed tissue, where they can persist for extended periods [244]. This persistent presence may provoke sustained local foreign-body reactions and chronic inflammation, thereby disrupting the normal tissue repair process. More importantly, accumulated nanoparticles may compromise collagen deposition and cross-linking either by inducing oxidative stress or through direct physical interference, undermining the long-term quality of healed tissue [245]. Second, the uptake of nanoparticles by sensory nerve endings or dermal fibroblasts represents an underappreciated risk. Nanoparticles can enter dermal fibroblasts via nonspecific endocytosis and impair their proliferation, migration, and ECM synthesis, consequently damaging granulation tissue formation [245]. Similarly, nanoparticles taken up by regenerating sensory nerve endings may exert neurotoxic effects or physically impede axonal growth, potentially causing abnormal sensation or impaired nerve regeneration, even damage to the central nervous system because of sensory nerve endings transmission, and that is an area that remains virtually unexplored [246,247]. Third, the long-term impact of nanoparticles on skin remodeling and scar formation merits careful consideration. Because nanomaterials can persist in the wound site for weeks or longer, their ongoing physical presence or sustained ion release may disrupt the remodeling equilibrium during the later stages of healing, leading to an imbalance in collagen type ratios or excessive deposition [248]. Such interference can precipitate pathological scars such as hypertrophic scars or result in compromised biomechanical properties of the healed skin, including reduced tensile strength. To address this knowledge gap, relevant assessment methods have been explored. For example, a study using advanced porcine skin models combined with dermal open-flow microperfusion demonstrated that AgNPs exhibit negligible penetration through both intact and damaged skin, with no substantial local inflammatory response [249]. Conducted within the EU’s “Safety and Sustainable Design” framework, this work emphasizes early integration of safety and sustainability into material innovation. However, such studies remain scarce, and standardized assessment protocols and long-term clinical data are currently lacking to define nanomaterial biodegradability, in vivo fate, and chronic toxicity. Future research must therefore incorporate long-term safety and scar quality as core endpoints and promote the development of standardized regulatory guidelines specifically for nanomaterial wound products.

4. Regulatory ambiguity. Nanomaterial-based wound therapies frequently occupy a regulatory gray zone, as their classification as drugs, medical devices, or combination products hinges on their primary mechanism of action and intended use [250]. In the United States, wound care products are regulated by different FDA centers according to their primary mode of action: devices fall under the purview of the Center for Devices and Radiological Health (CDRH), whereas products containing drugs, biologics, or growth factors may require evaluation by the Center for Drug Evaluation and Research (CDER) or the Center for Biologics Evaluation and Research (CBER) [251,252]. Combination products, for example, hydrogel dressings incorporating both antimicrobial nanoparticles and growth factors, encounter even more complex regulatory requirements, potentially necessitating compliance with multiple approval standards. Globally, firm regulatory guidelines specifically tailored to nanomedicines remain absent, largely owing to insufficient knowledge of nanomaterial properties and a lack of standardized testing methodologies [253]. This regulatory uncertainty prolongs the approval process, elevates development costs, and discourages investment in nanomedicine translation.

### Design parameters for deep penetration and local retention

Beyond biochemical and regulatory obstacles, the DW bed presents a formidable physical barrier that severely restricts nanomaterial access to the underlying viable tissue. This barrier is composed of 3 interconnected layers: (a) a superficial layer of avascular, tightly adherent necrotic tissue and fibrinous slough; (b) a dense, crosslinked ECM enriched in collagen types I and III, fibronectin, and proteoglycans, further stiffened by the accumulation of AGEs; and (c) biofilm-encased bacterial communities embedded within a self-produced extracellular polymeric substance. Collectively, these structures form a diffusion-limiting shell that traps topically applied nanoparticles at the wound surface, preventing their penetration to the metabolically active cells residing in deeper tissue compartments. Moreover, even when nanoparticles succeed in crossing this barrier, they are subject to rapid clearance driven by continuous wound exudate flow and lymphatic drainage, which together remove both soluble and particulate agents from the wound bed.

To overcome these physical barriers, the rational design of nanomaterials must carefully balance several interdependent physicochemical parameters. First, particle size serves as a primary determinant of diffusivity. Particles smaller than 100 nm can navigate the collagen meshwork of the ECM; however, their small dimensions also render them susceptible to rapid lymphatic washout from the wound bed [254,255]. In contrast, larger particles (>200 nm) exhibit prolonged tissue retention but encounter steric hindrance that restricts their interstitial movement. Size-switchable systems, for example, those that degrade from larger aggregates into smaller fragments upon exposure to wound-specific stimuli, offer a promising compromise by combining the retention advantages of large particles with the penetration capacity of small ones. Second, particle shape substantially influences transport through the porous ECM. Rod-shaped or filamentous nanoparticles diffuse more readily than their spherical counterparts, likely owing to their anisotropic geometry, which facilitates alignment with matrix fibers and reduces steric obstruction. Third, surface charge governs electrostatic interactions with the anionic ECM components. Cationic particles bind avidly to negatively charged matrix constituents and are therefore well retained at the wound surface; however, this strong binding impedes their penetration into deeper tissue layers. Anionic particles diffuse more freely through the ECM but are cleared more rapidly by exudate flow. Charge-reversal strategies, in which nanoparticles are engineered to be neutral or slightly negatively charged at physiological pH and become positively charged upon reaching the acidic microenvironment of the wound, can balance deep penetration with efficient cellular internalization [256]. Fourth, penetration-enhancing motifs, such as collagenase or hyaluronidase codelivered from nanocarriers, can transiently degrade the dense ECM to create diffusion channels. The therapeutic utility of this approach depends critically on the spatiotemporal control of enzyme release, for instance, through the use of MMP-sensitive linkers, to avoid compromising overall tissue integrity. An alternative strategy employs cell-penetrating peptides (e.g., TAT and RGD) that facilitate transcytosis across cellular barriers without the need for ECM degradation. Finally, resistance to exudate-driven washout and lymphatic drainage can be substantially improved through surface engineering. Mucoadhesive coatings, such as polydopamine, ECM-binding domains like collagen-binding peptides, or encapsulation within a hydrogel matrix can anchor the nanoparticles at the delivery site and sustain their therapeutic release. Importantly, these design parameters do not act in isolation but synergistically influence the overall transport behavior, and their integrated optimization is essential to achieve effective delivery into the hostile physical environment of the DW.

It is important to emphasize that these design parameters do not function independently but rather in a synergistic manner. For example, a small, rod-shaped, charge-reversible nanoparticle that codelivers collagenase within a hydrogel depot can, in principle, simultaneously achieve deep penetration into the wound bed and sustained therapeutic release. Quantitative depth-profiling studies employing ex vivo human wound tissue are therefore urgently needed to empirically validate these design strategies and to enable rational cross-platform comparisons.

### Design trade-offs in multifunctional nanosystems

The preceding sections illustrate a prevailing paradigm in nanomedicine for DW healing: the progressive integration of multiple therapeutic functions, antibacterial, glucose-lowering, antioxidant, pro-angiogenic, and immunomodulatory, into a single nanoplatform. While this “all-in-one” strategy carries the conceptual appeal of coordinated, multitargeted microenvironmental reprogramming, it also introduces a series of interconnected trade-offs that warrant critical scrutiny.

First, the escalation of functional complexity directly amplifies formulation intricacy and batch-to-batch variability. Each additional component, including enzymes, metal ions, photosensitizers, or therapeutic cargoes, must be incorporated with precise stoichiometric control and tailored release kinetics. As the number of constituents increases, even minor deviations in individual parameters, including loading efficiency, scaffold porosity, or degradation rate, can propagate cumulatively, resulting in substantial heterogeneity in overall therapeutic performance. This inherent sensitivity poses formidable challenges for GMP-compliant manufacturing, where reproducible product specifications and consistent biological activity are mandatory, and each functional element requires its own set of validated analytical methods for identity, purity, and potency.

Second, multifunctional systems substantially broaden the spectrum of toxicological concerns. Each constituent material possesses distinct biodistribution, clearance, and immunogenicity profiles, and their degradation by-products may interact in ways that are difficult to predict. For example, metal ions released from one functional module can perturb the catalytic activity of codelivered enzymes through competitive binding or redox interactions, or they may elicit off-target inflammatory responses by activating innate immune receptors. Moreover, the long-term tissue accumulation of multiple degradation products, particularly those derived from nonbiodegradable inorganic components, remains inadequately characterized in most preclinical studies, yet carries substantial safety implications for clinical translation.

Third, the regulatory pathway becomes considerably more complex for multifunctional nanosystems. A single platform that simultaneously acts as an antimicrobial agent (device-like), a drug carrier (drug-like), and a biological modulator (biologic-like) resists unambiguous classification by the FDA, because the determination of primary mode of action makes the criterion used by the FDA to assign a product to a specific center for review becomes ambiguous when multiple therapeutic mechanisms operate concurrently. This ambiguity may necessitate multicenter evaluation involving the CDRH, the CDER, and the CBER, substantially prolonging approval timelines and increasing regulatory uncertainty [251,252]. Such complexity disproportionately burdens academic spin-offs and small enterprises that often lack the dedicated regulatory expertise and financial resources required to navigate these overlapping jurisdictional frameworks.

Fourth, the assumption that “more functions are better” remains largely untested empirically. It is not yet established whether a 5-function nanosystem confers clinically meaningful advantages over a carefully optimized 2-function analogue. Given the highly interconnected nature of the pathological network in DWs, targeting a single critical driver, such as biofilm eradication or the neutralization of ROS, may secondarily alleviate downstream inflammation and angiogenesis impairment through the disruption of self-reinforcing feedback loops. If so, the addition of separate functional modules to address these secondary outcomes may be redundant rather than synergistic. This uncertainty underscores the need for a more disciplined, mechanism-guided approach to nanoplatform design.

To navigate these trade-offs, we propose 3 guiding principles. First, a modular architecture should be adopted, in which individual functional components are independently optimized and can be assembled in a facile, plug-and-play manner. This approach confines the complexity of each module, simplifies troubleshooting, and facilitates the substitution or upgrading of individual functions without necessitating a complete redesign of the entire nanosystem. Second, orthogonal characterization pipelines must be established that extend well beyond acute cytotoxicity assays. These should encompass rigorous assessment of batch-to-batch consistency, comprehensive degradation profiling under clinically relevant wound conditions, and tiered biocompatibility evaluations that systematically address subacute and chronic toxicity, immunogenicity, and the long-term fate of degradation products. Third, comparative efficacy studies should be mandated to directly contrast multifunctional systems with their simpler, function-reduced counterparts, thereby empirically quantifying the incremental therapeutic benefit gained from each additional layer of complexity. Such head-to-head comparisons are essential to distinguish genuine synergy from functional redundancy and to justify the added manufacturing and regulatory burden.

### Redefining therapeutic success and identifying the most promising strategies

At present, the vast majority of preclinical studies and even clinical trials rely on time to complete wound closure as the primary endpoint, typically defined as over 95% or 100% re-epithelialization. While this metric is objective and clinically informative, it is increasingly acknowledged to be an incomplete measure of genuine wound resolution. In the diabetic context, healing must be evaluated not solely by the speed of epithelial coverage but also by the quality and functional integrity of the repaired tissue.

A comprehensive success metric should therefore incorporate at least 4 additional dimensions: (a) restoration of skin barrier function, as assessed by transepidermal water loss and stratum corneum integrity; (b) sensory reinnervation, as evaluated by intraepidermal nerve fiber density and quantitative sensory testing, a parameter of particular relevance given that peripheral neuropathy is a hallmark of diabetic complications; (c) biomechanical competence, as measured by tensile strength and elasticity of the healed skin, which collectively determine resistance to recurrence under mechanical loading; and (d) functional and aesthetic outcomes, including scar extent, pigmentation, and tissue pliability, which profoundly influence patient quality of life.

Addressing these dimensions necessitates a transition from contraction-prone rodent wound models, which do not faithfully recapitulate human healing, to porcine or humanized skin equivalents capable of evaluating these endpoints. Concurrently, clinical trials must adopt composite endpoints that incorporate validated scar scales, noninvasive skin function diagnostics, neurological assessments, and patient-reported outcomes, with extended follow-up beyond 12 weeks to capture late-stage scar maturation and recurrence [257]. Such a paradigm shift would incentivize the design of genuinely reparative nanotherapies that restore structural, sensory, and mechanical integrity, rather than merely accelerating epithelial coverage, thereby improving long-term patient outcomes and reducing the unacceptably high recurrence rates that continue to undermine current DW management. Based on a critical evaluation of therapeutic functions, design trade-offs, and translational feasibility, we identify the following frontrunners. Firstly, the most readily translatable strategy is the mild PTT hydrogel incorporating silver or zinc complexes. Its translational advantage derives from the existing FDA clearance of silver-based dressings, a well-established hydrogel manufacturing infrastructure, and a regulatory pathway that often qualifies it as a device–drug combination, thereby streamlining the approval timeline. This approach robustly targets biofilm without imposing additional oxidative injury, positioning it as the safest candidate for near-term clinical adoption. Secondly, the most biologically promising strategy is the tissue-engineered MSC-EVs platform. Although currently constrained by scalability hurdles, EVs intrinsically orchestrate the entire healing cascade—from inflammation resolution to functional angiogenesis—in a physiologically coordinated manner. Once standardized, GMP-compliant, large-scale EV production is achieved, their superior bioactive cargo and low immunogenicity are likely to outperform synthetic multifunctional nanoparticles for late-stage tissue regeneration. Finally, the most forward-looking, high-reward strategy is GOx-nanozyme cascade reactor equipped with feedback-responsive elements and the probiotic living system. Despite current challenges in cold-chain logistics and complex regulatory oversight, these systems uniquely offer the potential to establish a self-regulating “closed loop” that autonomously adapts to real-time fluctuations in glucose and ROS levels, thereby representing the ultimate realization of the de novo design philosophy.

## Conclusions

Nanotechnology holds transformative potential for the treatment of DWs by enabling precise, multifunctional modulation of the pathological wound microenvironment. Through tailored material designs, including metal-based nanoparticles, enzyme-loaded nanosystems, EVs, and stimulus-responsive hydrogels, nanomaterials can simultaneously address infection, hyperglycemia, oxidative stress, impaired angiogenesis, and chronic inflammation. These integrated strategies move beyond conventional palliative care to actively reprogram the wound bed toward a healing-competent state, as demonstrated in numerous preclinical studies. By leveraging nanoscale properties for targeted, sustained, and stimuli-responsive delivery, these platforms offer a versatile toolkit to overcome the complex, interdependent barriers that characterize DWs healing.

Looking forward, several key directions will shape the evolution and clinical translation of nanomaterial-based therapies for DWs. First, the design of multifunctional and hierarchically structured nanosystems that combine antibacterial, antioxidant, immunomodulatory, and pro-angiogenic actions within a single platform will be essential to address the multifactorial nature of chronic wounds. Second, the development of intelligent, feedback-responsive systems capable of sensing and adapting to dynamic wound biomarkers (e.g., pH, ROS, and enzyme levels) could enable autonomous, context-dependent therapeutic release, maximizing efficacy while minimizing off-target effects. Third, overcoming translational barriers, including scalability under GMP standards, long-term biocompatibility, and regulatory pathways, will be critical to move promising nanotherapies from bench to bedside. Collaborative efforts among material scientists, clinicians, and regulatory bodies will be needed to establish standardized evaluation frameworks and accelerate clinical adoption.

Based on the current state of the field, we propose a series of forward-looking strategic shifts: nanoplatform design must evolve from functional stacking toward coordinated sequentiality through microenvironment-sensing smart systems that enable truly adaptive therapy; immunomodulation should transcend the simplistic M1/M2 dichotomy to restore the spatiotemporal coordination of the entire immune network and avoid aberrant fibrosis; translational challenges necessitate the early integration of clinically relevant comorbidity models, scalable GMP-compliant synthesis, and proactive regulatory classification planning; and a multidimensional efficacy framework encompassing barrier function, sensory reinnervation, biomechanical strength, and long-term recurrence resistance must replace time-to-closure as the core benchmark for therapeutic success, thereby ensuring that structural, functional, and aesthetic repairs are simultaneously achieved to meet the fundamental needs of patients with DWs.

Furthermore, recent advances in AI are beginning to inform both nanomaterial design and wound management. In the domain of nanomedicine development, machine learning models are increasingly employed to optimize nanoparticle formulation parameters. For instance, Rezvantalab et al. [258] apply 4 different machine learning techniques to predict the size, encapsulation efficiency, and drug loading of PLGA-based nanoparticles, identifying molecular weight and lactide-to-glycolide ratio as the most critical determinants of nanoparticle performance. Similarly, the AGILE platform, a deep learning-driven approach, successfully streamlines the development of ionizable lipids for mRNA delivery by enabling rapid in silico screening and selection from extensive lipid libraries [259]. In parallel, deep learning-based wound image analysis shows promise for the early prediction of healing outcomes in DWs. Spinazzola et al. [260] develop predictive models using deep neural networks and machine learning algorithms on a dataset of 1,766 diabetic foot wounds, achieving 80% accuracy in healing prediction. Although these studies focus either on nanomaterials or on DWs in isolation, they collectively establish a solid foundation for AI-assisted research specifically targeting nanomaterials for DW healing.

Nevertheless, the clinical translation of AI-integrated nanomedicine faces substantial hurdles. These include the scarcity of standardized, high-quality datasets with consistent annotation protocols, the “black-box” nature of deep learning models that challenges regulatory interpretability requirements, and a general lack of prospective clinical validation in diverse patient populations [261]. Overcoming these barriers will require coordinated efforts to establish shared data repositories, develop explainable AI frameworks, and design rigorous prospective validation studies.

In summary, this review moves beyond a descriptive catalog of nanomaterial functions to propose a paradigm-shifting “de novo design” framework for DW therapy. By mandating a hierarchical, temporal, and systems-level approach from the outset, this framework directly addresses a fundamental shortcoming of conventional nanomedicine design: the tendency to treat pathological factors as parallel, independent targets rather than as nodes within a causally structured network. We provide a conceptual and practical roadmap to guide the rational development of next-generation nanomedicines that are not merely multifunctional but genuinely synergistic and context-aware. We argue that this deliberate transition from a “function-stacking” philosophy to one of “sequence-programming” is essential to dismantle the self-reinforcing vicious cycle of the DW microenvironment and to achieve durable, high-quality healing.

## Data Availability

This review is based on the analysis of data extracted from previously published studies, all of which are cited within the article. No new primary data were generated for this review.
